# Peters plus syndrome mutations affect the function and stability of human β1,3-glucosyltransferase

**DOI:** 10.1016/j.jbc.2021.100843

**Published:** 2021-05-28

**Authors:** Ao Zhang, Aarya Venkat, Rahil Taujale, James L. Mull, Atsuko Ito, Natarajan Kannan, Robert S. Haltiwanger

**Affiliations:** 1 Complex Carbohydrate Research Center, University of Georgia, Athens, Georgia, USA; 2 Department of Biochemistry and Molecular Biology, University of Georgia, Athens, Georgia, USA; 3 Institute of Bioinformatics, University of Georgia, Athens, Georgia, USA

**Keywords:** glycosyltransferase, genetic disease, glycobiology, glycoprotein secretion, enzyme catalysis, Peters plus syndrome, B3GLCT, *O*-fucose, thrombospondin type-1 repeats, ADAMTS, A Disintegrin-like And Metalloprotease with Thrombospondin type-1 repeats, B3GLCT, β1,3-glucosyltransferase, B3GNT2, β1,3-N-acetylglucosaminyltransferase 2, CAZy, Carbohydrate Active enZYmes, ECM, extracellular matrix, G-loop, glycine-rich loop, GT, glycosyltransferase, MFNG, Manic fringe, PDB, Protein Data Bank, PTRPLS, Peters plus syndrome, RMSD, root-mean-square deviation of atomic positions, TSR, thrombospondin type-1 repeats, WT, wild type

## Abstract

Peters Plus Syndrome (PTRPLS OMIM #261540) is a severe congenital disorder of glycosylation where patients have multiple structural anomalies, including Peters anomaly of the eye (anterior segment dysgenesis), disproportionate short stature, brachydactyly, dysmorphic facial features, developmental delay, and variable additional abnormalities. PTRPLS patients and some Peters Plus-like (PTRPLS-like) patients (who only have a subset of PTRPLS phenotypes, have mutations in the gene encoding β1,3-glucosyltransferase [*B3GLCT*]). B3GLCT catalyzes the transfer of glucose to *O*-linked fucose on thrombospondin type-1 repeats. Most B3GLCT substrate proteins belong to the ADAMTS superfamily and play critical roles in extracellular matrix. We sought to determine whether the PTRPLS or PTRPLS-like mutations abrogated B3GLCT activity. B3GLCT has two putative active sites, one in the N-terminal region and the other in the C-terminal glycosyltransferase domain. Using sequence analysis and *in vitro* activity assays, we demonstrated that the C-terminal domain catalyzes transfer of glucose to *O*-linked fucose. We also generated a homology model of B3GLCT and identified D421 as the catalytic base. PTRPLS and PTRPLS-like mutations were individually introduced into B3GLCT, and the mutated enzymes were evaluated using *in vitro* enzyme assays and cell-based functional assays. Our results demonstrated that PTRPLS mutations caused loss of B3GLCT enzymatic activity and/or significantly reduced protein stability. In contrast, B3GLCT with PTRPLS-like mutations retained enzymatic activity, although some showed a minor destabilizing effect. Overall, our data supports the hypothesis that loss of glucose from B3GLCT substrate proteins is responsible for the defects observed in PTRPLS patients, but not for those observed in PTRPLS-like patients.

Peters plus syndrome (PTRPLS OMIM #261540) is a rare, autosomal recessive, congenital disorder of glycosylation that is characterized by multiple structural defects, including Peters anomaly of the eye (anterior eye chamber segment dysgenesis), disproportionate short stature, brachydactyly, craniofacial defects (including cleft lip/palate and broadened forehead), developmental delay, and other systematic abnormalities at variable penetrance (1). These patients carry intronic and/or exonic mutations in the gene encoding β1,3-glucosyltransferase (*B3GLCT*, formerly *B3GALTL*), which can be homozygous or compound heterozygous (1, 2, 3). Most mutations in PTRPLS cause splicing or frameshift defects that can disrupt the transcription and/or translation of *B3GLCT* and are predicted to be loss of function. However, there are also several missense and nonsense mutations in *B3GLCT* (Fig. 1*A*). The missense mutations result in amino acid (aa) changes: D349N, G393E, G394E (Fig. 1*A*). PTRPLS-like patients have Peters anomaly and a subset of the additional phenotypes found in PTRPLS patients (3). While most PTRPLS-like patients have no mutations in *B3GLCT*, suggesting that mutations in other genes are causative for the phenotypes observed, some PTRPLS-like patients have heterozygous missense mutations in *B3GLCT*: T179S, V245M, R337H, and Q457R (personal communication, Dr Elena V. Semina, Medical College of Wisconsin) (Fig. 1*A*) (3). It is not clear whether these PTRPLS-like mutations contribute to the phenotypes observed in these patients. This raises a question regarding whether these PTRPLS or PTRPLS-like mutations affect B3GLCT function and/or stability. Two nonsense mutations were also identified from PTRPLS patients—Y366∗ (2) and R412∗ (3) (Fig. 1*A*). B3GLCT contains an REEL motif at the C-terminus of the protein (Fig. 1*A*) that serves as a KDEL-like motif, retaining B3GLCT within the endoplasmic reticulum (ER) (4, 5). The premature termination from Y366∗ and R412∗ results in deletion of the REEL motif, raising the question of whether these two mutations cause mislocalization of the truncated B3GLCT.

**Figure 1:**
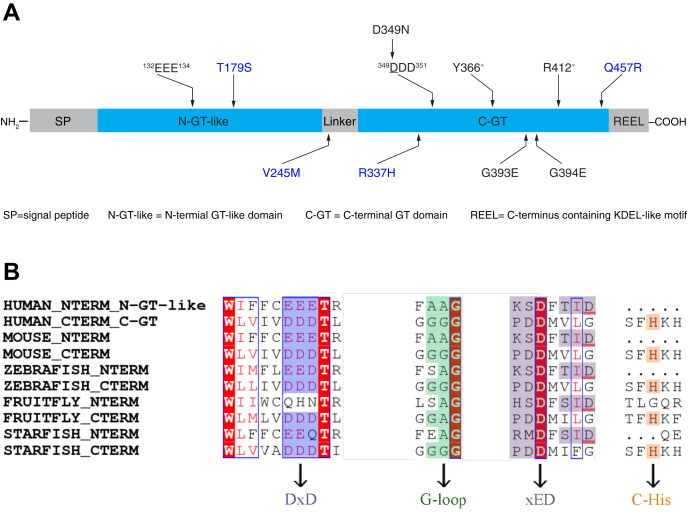
**Human B3GLCT contains two putative GT domains, and PTRPLS/PTRPLS-like mutations are predominantly localized in the C-GT domain.***A*, domain map of human B3GLCT with schematic distribution of PTRPLS (*black*) and PTRPLS-like (*blue*) mutations throughout the protein. Two putative DxD motifs, ^132^EEE^134^ and ^349^DDD^351^, are highlighted with *arrows*. *B*, common GT-A fold elements (6) identified from the multiple sequence alignment of the N-GT-like and C-GT domains from B3GLCT shown in Figure S1. Human (UNIPROT Q6Y288), mouse (UNIPROT Q8BHT6), zebrafish (UNIPROT A0A068F9P7), fruit fly (UNIPROT X2JDC2), and starfish (NCBI REFSEQ XP_038056415.1). Sequences were aligned with the MAFFT software/algorithm. *Red background* highlights regions of 100% sequence identity within the shown sequences; *red letters* indicate regions of 100% sequence similarity; *purple boxes* highlight DxD motifs; *green boxes* highlight G-loop; *gray boxes* highlight predicted xED motifs (note that two potential xED motifs are highlighted for N-GT-like domains); *orange boxes* highlight C-His.

B3GLCT belongs to the glycosyltransferase (GT)-31 family based on the Carbohydrate Active enZYmes (CAZy) classification (7). Currently, there are over 30 vertebrate subfamilies in the GT31 family (8), but structures of only two have been solved—mouse Manic Fringe (MFNG) (9) and human β1,3-N-acetylglucosaminyltransfease 2 (B3GNT2) (10, 11). A recent phylogenetic study demonstrated that B3GLCT is a unique member of the GT31 family because it harbors two putative GT domains, which are more similar to each other than the rest of the members in the GT31 family (8). Heinonen *et al.* (4) initially discussed that there are two “DxD” motifs in human B3GLCT: ^132^EEE^134^ in the N-terminal region (originally annotated as a stem region) and ^349^DDD^351^ in the C-terminal GT domain (C-GT) (Fig. 1*A*). The “DxD” motif marks the active site of GT-A fold glycosyltransferases and is responsible for the binding of nucleotide sugar donors and the chelation of divalent cation cofactors for catalysis (6). Kozma *et al.* (12) demonstrated that mutation of ^349^DDD^351^ to ^349^ADD^351^ or ^349^ADA^351^ significantly reduced *in vitro* B3GLCT enzymatic activity, but whether the N-terminal domain containing ^132^EEE^134^ is catalytically active is unknown. Nonetheless, the presence of two distinct domains in B3GLCT raises the question of whether PTRPLS is caused by loss of function of the N-terminal or C-terminal domains.

B3GLCT catalyzes the transfer of glucose (Glc) to *O*-linked fucose (*O*-Fuc) on thrombospondin type-1 repeats (TSRs), forming a unique glucose-β1,3-fucose disaccharide (GlcFuc) (5, 12). Protein *O*-fucosyltransferase 2 (POFUT2) catalyzes the addition of fucose (Fuc) to the hydroxyls of serine (Ser) or threonine (Thr) within the consensus sequence C-X-X-(S/T)-C of properly folded TSRs (13, 14). Each TSR is a small protein domain of 50–60 aa with six conserved cysteines that form three disulfide bonds (15, 16). TSRs exist in two groups depending on the disulfide bonding pattern. Thus, the consensus sequence occurs between the first and the second cysteine (C^1^-X-X-(S/T)-C^2^) of group 1 TSRs and the second and the third cysteine (C^2^-X-X-(S/T)-C^3^) of group 2 TSRs (16). Forty-nine human proteins contain TSR motifs with the consensus sequences (17), and half of these POFUT2/B3GLCT substrate proteins belong to the ADAMTS (A Disintegrin-like And Metalloprotease with Thrombospondin type I repeats) superfamily (18). Essentially all TSRs with the POFUT2 consensus sequence in POFUT2/B3GLCT substrate proteins that have been analyzed in detail are modified at high stoichiometry with the glucose-β1,3-fucose disaccharide (3, 19, 20, 21, 22, 23, 24, 25, 26, 27, 28). This suggests that B3GLCT modifies any TSR that is modified with *O*-fucose.

The ADAMTS and noncatalytic ADAMTS-like proteins are localized in the extracellular matrix (ECM) and have critical roles in organogenesis, tissue organization, and cell signaling during developmental processes (29). Mutations in several ADAMTS/ADAMTS-like proteins cause congenital disorders in humans and developmental defects in mice. For instance, mutations in *ADAMTSL2* cause geleophysic dysplasia, where patients display short stature, short tubular bones, thick skin, and cardiopulmonary abnormalities (30). Elimination of *Adamts9* in mice results in early embryonic lethality in mice. Interestingly, *Pofut2* knockout mice display the same phenotype, suggesting that ADAMTS9 requires addition of *O*-fucose to its TSRs for proper function (31). Heterozygous *Adamts9* mice show an anterior segmentation dysgenesis in the eyes, as seen in PTRPLS and PTRPLS-like patients (24). *Adamts20*-null mice show a white spotting defect and a high degree of hydrocephalus (26). Significantly, *B3glct* knockout mice phenocopy several defects seen in PTRPLS patients (craniofacial abnormalities, bone growth defects) but also show white spotting and hydrocephalus, indicating that ADAMTS20 is a biologically relevant B3GLCT substrate that requires addition of glucose to be fully functional (26).

A growing body of data supports the concept that POFUT2 and B3GLCT participate in a quality control pathway for the folding of TSRs (16). Both of these enzymes are located in the endoplasmic reticulum (ER), and deleting them results in secretion defects in a protein-specific manner. For instance, secretion of a portion of ADAMTS9 (TSRs2-8) is completely inhibited in *POFUT2*^−/−^ HEK293T cells, but only reduced by 20% in *B3GLCT*^−/−^ cells compared with wild type (WT) (26). Thus, the embryonic lethality of *Pofut2* knockout mice can be explained by loss of secretion of ADAMTS9. In contrast, ADAMTS20 TSR2-8, with almost identical TSR domain structures compared with ADAMTS9, is completely dependent on B3GLCT for secretion, implying that the white spotting and hydrocephalus observed in *B3glct* knockout mice are due at least in part to loss of ADAMTS20 secretion (26). Taken together, these data raise the question of whether PTRPLS/PTRPLS-like mutations in B3GLCT will affect the secretion of a subset of POFUT2/B3GLCT substrate proteins by affecting the function and/or stability of B3GLCT.

To address the questions raised above, we analyzed the domain structure of B3GLCT in more detail and demonstrated that the C-GT domain of B3GLCT is responsible for the transfer of Glc to *O*-Fuc on TSRs. We then introduced PTRPLS/PTRPLS-like mutations individually to human B3GLCT and performed kinetic analysis of the WT and mutant forms of the enzyme using *in vitro* enzyme assays. We also investigated the function of the mutant enzymes *in vivo* with cell-based assays by investigating the ability of the mutants to rescue the secretion of ADAMTS20 TSR2-8 from *B3GLCT*^−/−^ cells. Our data suggested that PTRPLS mutations eliminated the function of B3GLCT, but PTRPLS-like mutants did not. We also generated a homology model of B3GLCT to predict the structural impact of these PTRPLS/PTRPLS-like mutations. Overall, our studies advanced our understanding of the molecular mechanisms that result in PTRPLS.

## Results

### B3GLCT has two putative GT domains, but only C-GT domain transferred Glc to *O*-Fuc on TSRs

We began by analyzing the domain distribution of human B3GLCT as illustrated in Figure 1*A*, including signal peptide (aa 1–68), N-terminal GT-like (N-GT-like) domain (aa 69–240), cysteine-rich linker domain (aa 241–263), C-terminal GT domain (C-GT) (aa 264–468), and C-terminus with ER retention signal REEL (aa 469–498). Both the N- and C-terminal domains have residues analogous to those found in GT-A fold enzymes, including a DxD motif, ^132^EEE^134^ and ^349^DDD^351^, respectively. The DxD motifs were conserved in vertebrates, but the N-terminal DxD motif was not conserved in fruit flies (Figs. 1*B* and S1). Sequence analysis showed that the C-GT domain also has a highly conserved G-loop (^393^GGG^395^), xED motif (^419^PDD^421^), and C-terminal Histidine (C-His, H463), all common GT-A fold elements (6) (Figs. 1*B* and S1). The G-loop confers flexibility, and the xED-Asp functions as a catalytic base in inverting GT-A fold enzymes (6). The putative N-terminal glycosyltransferase domain has some of these features including a potential G-loop (^187^AAG^189^) (Figs. 1*B* and S1). Other conserved features are difficult to predict, including the catalytic base (possibly D212 or D216) and the C-terminal His (Figs. 1*B* and S1).

**Figure 2:**
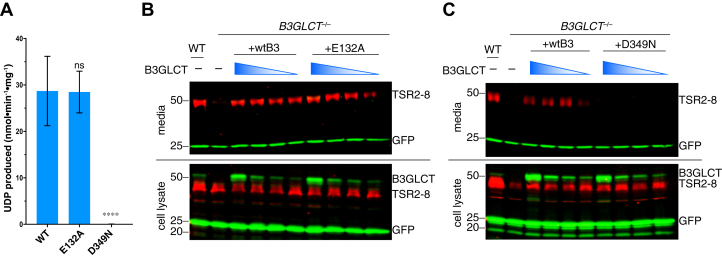
**The DxD motif of the C-GT domain is responsible for B3GLCT glucosyltransferase activity.***A*, *in vitro* enzyme activity assay (20 min) of WT, E132A mutant, and D349N mutant B3GLCTΔREEL with UDP-Glc as donor substrate (50 μM) and Fuc-*O*-TSR3 (25 μM) as acceptor substrate. Biological triplicates were performed with three different batches of enzymes. Error bars represent standard deviation, n = 3. Statistical analysis was performed with one-way ANOVA by comparing activities of the mutants to WT in Prism 7. ∗∗∗∗*p* < 0.0001; ns, not significant. *B* and *C*, plasmids encoding ADAMTS20 TSR2-8 (TSR2-8) and GFP were cotransfected into wild type (WT) and *B3GLCT* knockout (*B3GLCT*^−/−^) HEK293T cells. Rescue experiments were performed by cotransfection with a plasmid encoding full-length B3GLCT WT (wtB3), E132A mutant (*B*) or D349N mutant (*C*). Serial dilutions of B3GLCT-FL plasmids were performed starting with 0.24 μg of plasmids diluted 5-, 10-, and 20-fold. Media and cell lysates were analyzed by western blot probed with anti-Myc (*red*) to detect ADAMTS20 TSR2-8, anti-B3GLCT to detect endogenous or transfected B3GLCT (*green*, 50 kDa), and for transfection and loading control anti-GFP (*green*, 25 kDa).

To evaluate which domain is responsible for the glucosyltransferase activity, we first subcloned human B3GLCT with the REEL motif removed (B3GLCTΔREEL) to allow secretion to the medium for purification. We then mutated both DxD motifs (E132A and D349N) to abolish the ^132^EEE^134^ and ^349^DDD^351^ individually in B3GLCTΔREEL to further evaluate which of the two putative GT domains is responsible for catalytic activity. The D349N is also a PTRPLS mutation (3). We first used *in vitro* enzyme assays to analyze whether the E132A and D349N mutants could transfer glucose to *O*-fucosylated TSR3 from human thrombospondin-1 (Fuc-*O*-TSR3). After a 20 min transfer reaction (within the linear phase of the assay, Fig. S2), the D349N mutant completely lost catalytic activity, but E132A catalyzed the reaction at a similar level to the WT B3GLCTΔREEL (Fig. 2*A*). Since the N-terminal GT domain is not involved in transfer of glucose, and identification of some GT-A elements needed for catalysis is not clear, we termed it as “N-GT-like” in our domain map of human B3GLCT (Fig. 1*A*).

To further analyze the function of the domain mutants under physiological conditions, we tested the ability of the E132A and D349N mutants to rescue the secretion of ADAMTS20 TSR2-8 in *B3GLCT*^−/−^ cells. For these assays we used full-length human B3GLCT with the REEL motif intact (B3GLCT-FL) to retain transfected B3GLCT within the ER. ADAMTS20 TSR2-8 was secreted into the media from WT HEK293T cells but lost its secretion in *B3GLCT*^−/−^ cells (Fig. 2, *B* and *C*, media), as shown previously (26). The E132A mutant rescued the secretion of ADAMTS20 TSR2-8 similar to WT B3GLCT-FL (Fig. 2*B*, media), whereas D349N completely lost the ability to rescue (Fig. 2*C*, media). Both the E132A and D349N mutants were detected in cell lysate at comparable levels to transfected WT B3GLCT. Serial dilution of cotransfected enzyme plasmids allowed us to mimic the endogenous B3GLCT cellular level in WT HEK293T cells for better analysis (Fig. 2, *B* and *C*, cell lysate). Together, these data confirmed that the ^349^DDD^351^ in the C-GT domain, but not the ^132^EEE^134^ in the N-GT-like domain, is required for the biological function of B3GLCT. Together with the *in vitro* enzyme assays in Figure 2*A*, these data also demonstrate that the addition of glucose to ADAMTS20 TSR2-8 is required for secretion.

We further investigated whether the putative C-GT domain was able to function in the absence of the N-GT-like domain. We used PCR to generate plasmids with open reading frames (ORFs) beginning at three different positions throughout the linker due to the presence of three cysteine residues in this region (Fig. S3*A*). We generated two sets of these C-GT domains: one set without the REEL motif for expression and *in vitro* assays, the other with REEL intact to retain the truncated enzymes within the ER for cell-based assays. We failed to express the C-GT-ΔREEL constructs, so we employed our cell-based secretion assay as an alternative to analyze the C-GT constructs (with REEL intact) in cells (Fig. S3*B*). In all cases, we failed to detect the expression of the C-GT domains in the cell lysates, and none of these constructs rescued the secretion of ADAMTS20 TSR2-8 (Fig. S3*B*). We also attempted to express the N-GT-like domain, truncated at the same positions used for the C-GT domain (Fig. S3*C*). The N-GT-like domain was not detected in cell lysates or media, suggesting that the proteins are misfolded and cannot pass ER quality control. Together, these results suggest that the presence of both the N-GT-like and C-GT domains is likely required for proper folding of B3GLCT.

### PTRPLS mutations eliminated B3GLCT catalytic activity whereas PTRPLS-like mutations did not

**Figure 3:**
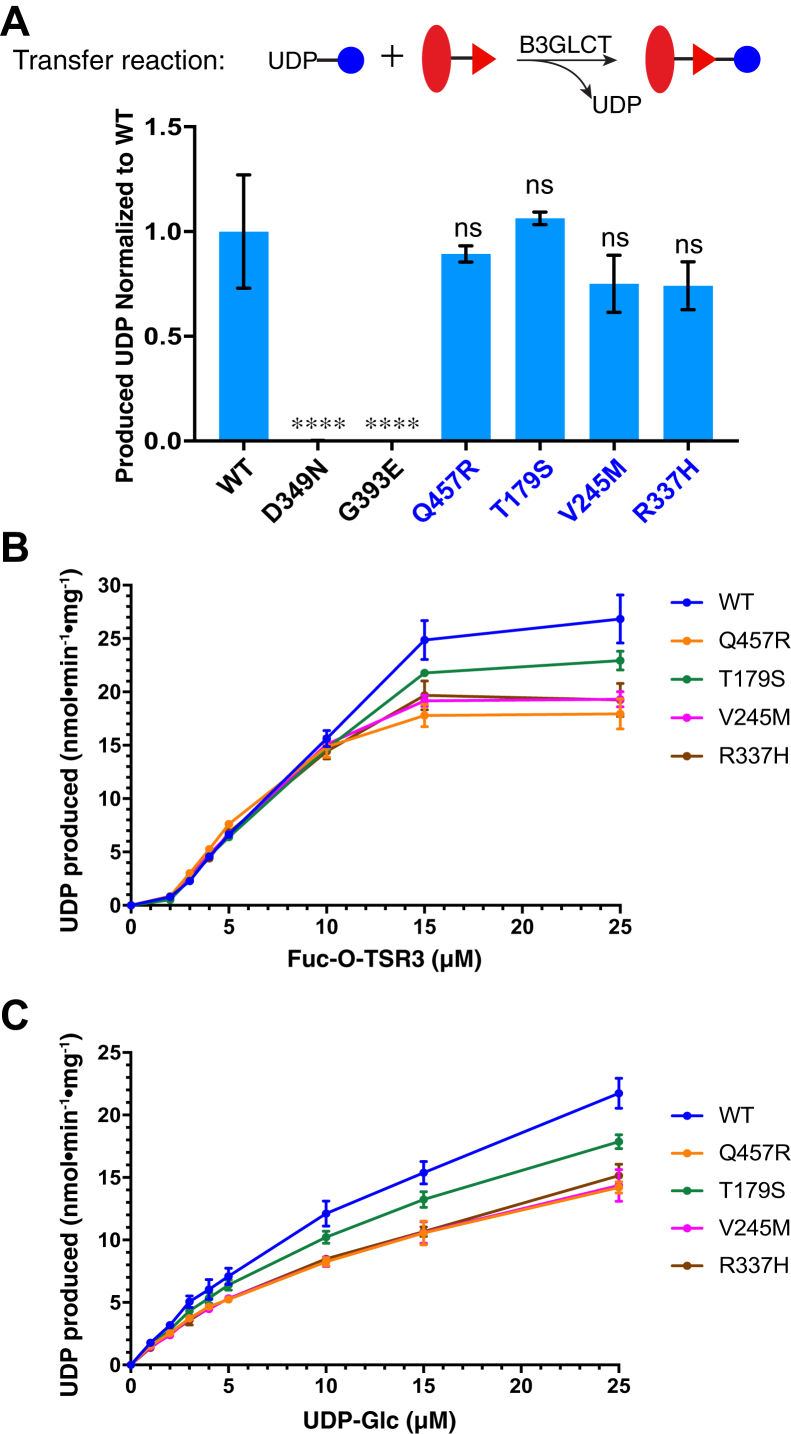
**PTRPLS mutants are not catalytically active, but PTRPLS-like mutants are.***A*, *top*, reaction catalyzed by B3GLCT for transfer Glc to Fuc-*O*-TSR3. *Solid blue circle*, glucose; *red oval*, TSR3; *red triangle*, fucose. *A*, *bottom*, *in vitro* enzyme assay (20 min) of WT, PTRPLS mutants (*black*), and PTRPLS-like mutants (*blue*). Calculated UDP produced by the mutants was normalized to the UDP produced by WT. Experiment was repeated in biological triplicates with three batches of purified enzymes. Error bars represent standard deviation, n = 3. Statistical significance was analyzed by comparison of the mutants to WT with one-way ANOVA analysis in Prism 7. ∗∗∗∗*p* < 0.0001; ns, not significant. *B* and *C*, substrate concentration dependent kinetics of WT and PTRPLS-like mutants with various Fuc-*O*-TSR3 (*B*) and UDP-Glc concentrations (*C*) (n = 3). Technical replicates with error bar show standard deviation for each point.

Figure 1*A* shows the distribution of all tested missense and nonsense mutations throughout the B3GLCT protein (PTRPLS in black and PTRPLS-like in blue). We introduced each mutation individually into human B3GLCTΔREEL and purified WT and all the mutant B3GLCTΔREEL from conditioned medium of HEK293T cells (Fig. S4). As shown in Figure 3*A*, PTRPLS D349N and G393E completely lost the ability to transfer Glc to Fuc-*O*-TSR3 in the 20 min transfer assays, whereas PTRPLS-like mutations retained WT catalytic activity. We performed kinetic analysis on the WT and PTRPLS-like mutant enzymes to examine the effects of these mutations in more detail. Interestingly, we observed a positive cooperativity of B3GLCT with increasing concentrations of the acceptor substrate Fuc-*O*-TSR3 (Fig. 3*B*). PTRPLS-like mutations retained this cooperativity with acceptor substrate (Fig. 3*B*). In contrast, UDP-Glc concentration-dependent assays showed a hyperbolic, Michalis–Menten enzyme behavior (Fig. 3*C*). Overall, PTRPLS-like mutants did not largely affect the enzymatic activity, and the shapes of the curves of the mutants were similar to those of WT B3GLCTΔREEL (Fig. 3, *B* and *C*). However, PTRPLS-like mutants slightly increased affinity of both donor and acceptor substrate by the measurement of reduced K_M_ and K_half_ respectively (Tables 1 and 2). PTRPLS-like mutants also slightly reduced the enzyme turnover number k_cat_ (Table 2), implying reduced catalytic efficiency for PTRPLS-like mutants.

**Table 1:** Fuc-*O*-TSP1TSR3 concentration-dependent kinetic analysis of WT and PTRPLS-like mutants

Kinetic parameter	WT	Q457R	T179S	V245M	R337H
V_max_ (nmol ⋅ min^−1^ ⋅ mg^−1^)	30.10	18.62	25.22	20.36	20.62
K_half_ (μM)	8.90	5.73	8.04	6.64	6.65

**Table 2:** UDP-Glucose concentration-dependent kinetic analysis of WT and PTRPLS-like mutants

Kinetic parameter	WT	Q457R	T179S	V245M	R337H
V_max_ (nmol ⋅ min^−1^ ⋅ mg^−1^)	43.21	23.56	32.44	24.63	27.73
K_M_ (μM)	25.47	17.39	20.94	18.74	21.89
k_cat_ (min^−1^)	2.42	1.32	1.82	1.38	1.55

### PTRPLS mutations prevented B3GLCT from rescuing secretion of ADAMTS20 TSR2-8, but PTRPLS-like mutations did not

**Figure 4:**
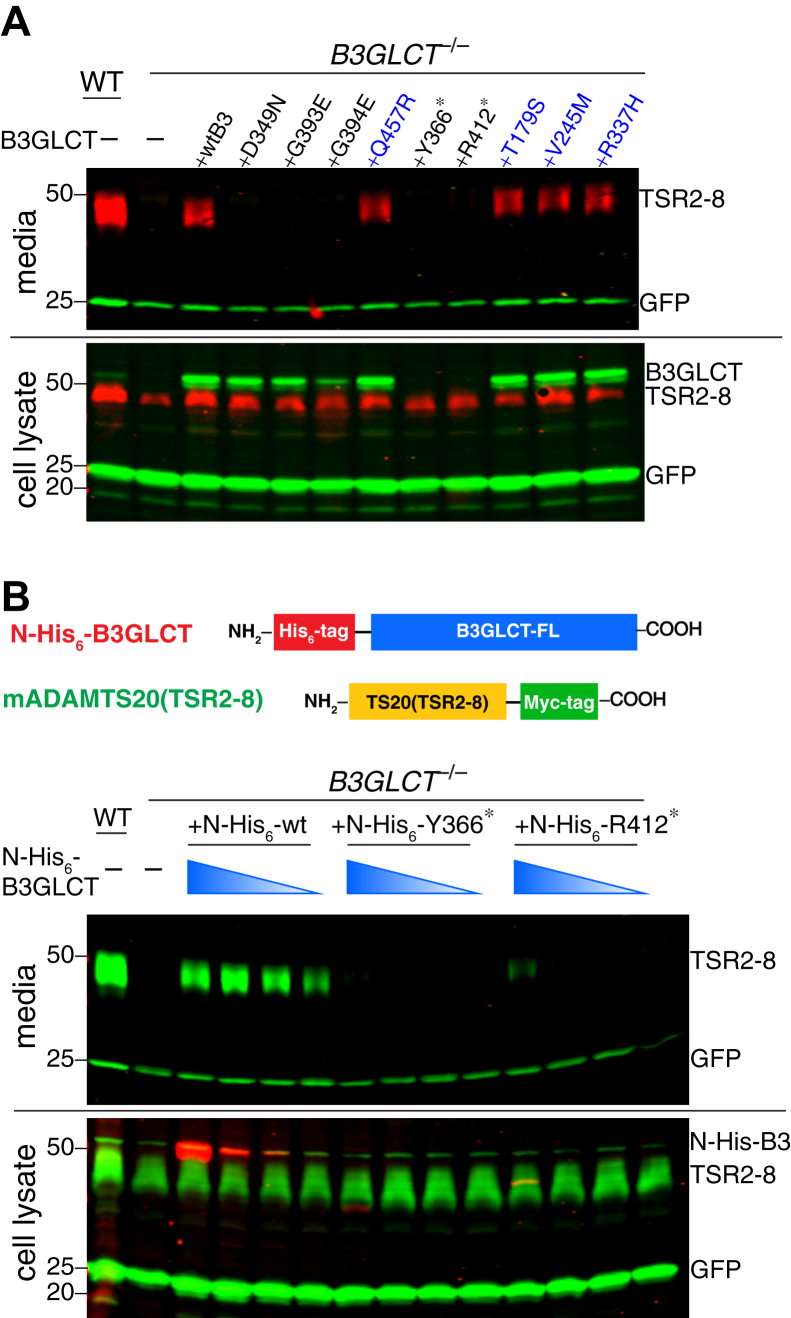
**PTRPLS, but not PTRPLS-like, mutations abolished the ability to rescue the secretion of ADAMTS20 TSR2-8 from *B3GLCT***^**−/−**^**cells**. *A*, plasmids encoding ADAMTS20 TSR2-8-Myc-His_6_ and GFP were cotransfected into WT or *B3GLCT*^−/−^ HEK293T cells. Rescue experiments were performed by cotransfection with plasmids encoding full-length WT, PTRPLS, or PTRPLS-like mutations in B3GLCT (B3GLCT). Media and cell lysates were analyzed by western blot probed with anti-Myc (*red*) to detect ADAMTS20 TSR2-8 (TSR2-8), anti-GFP for transfection and loading control (*green*, 25 kDa), and anti-B3GLCT for B3GLCT WT or mutants (*green*, 50 kDa). *B*, *top*, domain maps of N-His_6_-B3GLCT and ADAMTS20 TSR2-8-Myc constructs used in this experiment. *Bottom*, plasmids encoding ADAMTS20 TSR2-8-Myc and GFP were cotransfected into WT or *B3GLCT*^−/−^ HEK293T cells. Rescue experiments were performed by cotransfection with serial dilutions of plasmids encoding N-His_6_-B3GLCT, N-His_6_-Y366∗ mutant, or N-His_6_-R412∗ mutant. Serial dilutions of rescue plasmids were performed starting with 0.24 μg diluted 5-, 10-, and 20-fold. Media and cell lysates were analyzed by western blots probed with anti-Myc (*green*, 50 kDa) to detect ADAMTS20 TSR2-8-Myc (TSR2-8), anti-GFP (*green*, 25 kDa) for transfection and loading control, and anti-His_6_ (*red*, 50 kDa or less) for B3GLCT WT, Y366∗, and R412∗.

We were not able to express sufficient amounts of several PTRPLS mutants for *in vitro* enzymatic assays, including G394E, Y366∗ (as N-His_6_-Y366∗) and R412∗ (as N-His_6_-R412∗). Therefore, we utilized our cell-based assay as an alternative approach to evaluate the impact of the mutations on the function of B3GLCT by observing the ability of mutant enzyme to rescue ADAMTS20 TSR2-8 secretion from *B3GLCT*^−/−^ cells. Similar to the results in Figure 2, ADAMTS20 TSR2-8 was secreted into the media from WT HEK293T but not from *B3GLCT*^−/−^ cells (Fig. 4*A*, media). Cotransfected WT B3GLCT-FL (with REEL intact) or PTRPLS-like mutants were able to restore ADAMTS20 TSR2-8 secretion in *B3GLCT*^−/−^ cells (Fig. 4*A*, media). In contrast, PTRPLS mutants (D349N, G393E, G394E, Y366∗, and R412∗) failed to rescue ADAMTS20 TSR2-8 secretion in *B3GLCT*^−/−^ cells (Fig. 4*A*, media). We also observed reduced protein levels for G393E and G394E in cell lysates (Fig. 4*A*, cell lysate), suggesting that these mutants destabilized B3GLCT.

In Figure 4*A*, we did not detect Y366∗ and R412∗ in *B3GLCT*^−/−^ cell lysate as these two premature stop codon mutations eliminated the epitope for the anti-B3GLCT antibody (OriGene) that was used in these experiments. To overcome this issue, we inserted an N-terminal His_6_-tag immediately after the signal peptide of the human B3GLCT-FL plasmid construct and introduced Y366∗ and R412∗ individually into this N-His_6_-B3GLCT-FL plasmid. We used serial dilutions of cotransfected enzyme plasmids to mimic the endogenous B3GLCT enzyme level within the cells (Fig. 4*B*). The N-His_6_-Y366∗ mutant did not rescue the secretion of ADAMTS20 TSRS2-8 at all, whereas N-His_6_-R412∗ had a minimal level of rescue under the highest level of cotransfected enzyme plasmid (Fig. 4*B*, media). However, this minimal rescue was significantly lower than the secretion of ADAMTS20 TSR2-8 from WT cells or the rescue by WT B3GLCT-FL at the lowest plasmid amount. In the cell lysate, we only detected N-His_6_-Y366∗ and N-His_6_-R412∗ at the highest plasmid amount, and the protein levels for these two mutants were dramatically lower than WT N-His_6_-B3GLCT at the same plasmid amount (Fig. 4*B*, cell lysate). These data suggested that the Y366∗ and R412∗ mutations likely lead to destabilization of B3GLCT. We also performed secretion assays with serial diluted enzyme plasmids amount for all of the mutants (Fig. S5), and the results were consistent with Figure 4*A*. Similar to Y366∗ and R412∗, we also observed that G394E was only detectable in the cell lysate at the highest plasmid amount (Fig. S5*B*, cell lysate) presumably due to destabilization.

### PTRPLS G393E and PTRPLS-like Q457R destabilized B3GLCT

**Figure 5:**
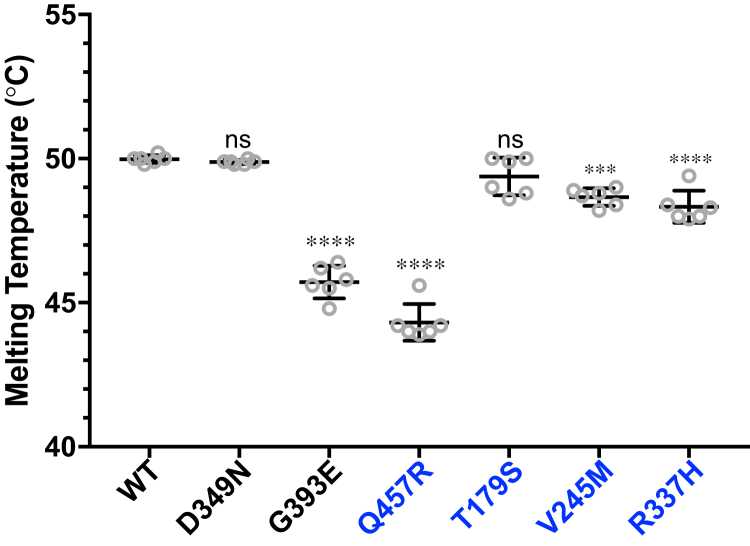
**PTRPLS G393E and PTRPLS-like Q457R mutants significantly destabilized B3GLCT.** Thermo shift assays with SYPRO Orange were employed to analyze the protein stabilities by measuring the melting temperatures of the proteins. Melting temperatures of PTRPLS mutants (*black*) and PTRPLS-like mutants (*blue*) were compared with the melting temperature of the WT (Fig. S5). Statistical analysis was performed with one-way ANOVA analysis in Prism 7. Error bars, standard deviation, n = 6 from two batches of purified enzymes with three replicates for each batch. ∗∗∗*p* < 0.001; ∗∗∗∗*p* < 0.0001; ns, not significant.

To analyze the impact of the mutations other than G394E, Y366∗, and R412∗ on the stability of B3GLCT, we measured the melting temperatures of the purified mutant enzymes with thermo shift assays using SYPRO Orange dye. Of all the tested mutants in this assay, G393E (PTRPLS mutant) and Q457R (PTRPLS-like mutant) decreased the melting temperature of B3GLCT by the largest extent, 4 °C and 6 °C respectively (Figs. 5 and S6). PTRPLS-like V245M and R337H slightly destabilized B3GLCT, with a 1 °C and 1.5 °C reduction of the melting temperature, respectively (Figs. 5 and S6).

### B3GLCT C-GT domain adopted a GT-A fold structure with catalytic base D421

**Figure 6:**
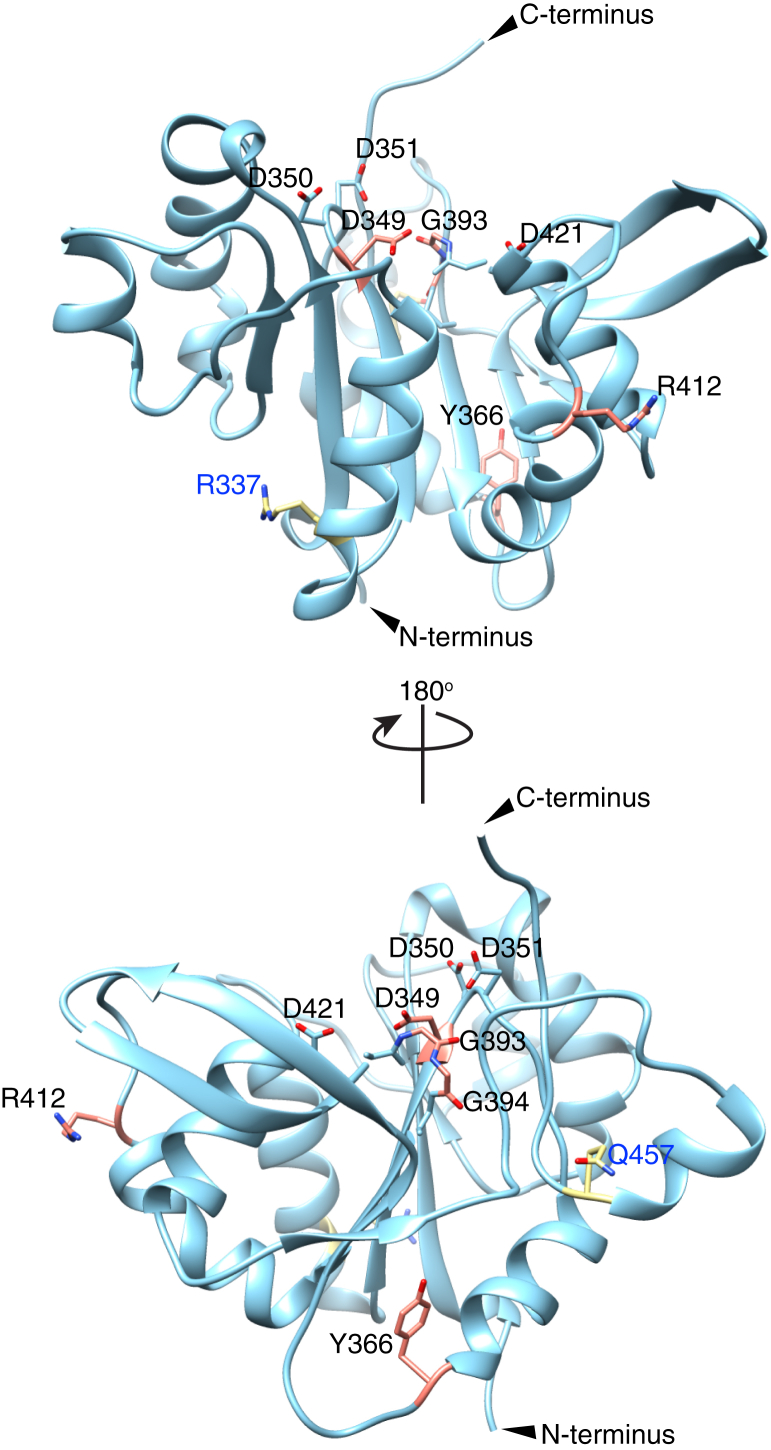
**Homology model revealed B3GLCT C-GT domain as a GT-A fold enzyme.** Structure of B3GLCT C-GT domain was generated by homology modeling and represented in ribbon (*light blue*). Key conserved residues are highlighted in *light blue*. PTRPLS mutations are highlighted in *salmon* and PTRPLS-like mutations are highlighted in *yellow*.

To predict the structural impact of the mutations on B3GLCT, we generated a homology model using SWISS MODEL. Submission of the full-length B3GLCT amino acid sequence resulted in models for the C-GT domain (aa 263–466) and the N-GT-like domain (aa 52–245) separately. Both models were based on the mouse Manic Fringe (MFNG) structure (PDB ID 2J0A) as template. Since the C-GT domain is responsible for the glucosyltransferase activity and accommodates the majority of the PTRPLS and PTRPLS-like mutations, we began our analysis with the C-GT domain model. As predicted from the sequence analysis (Figs. 1 and S1), the C-GT domain adopts a GT-A fold (Fig. 6). The C-GT ^349^DDD^351^ and ^393^GGG^395^ motifs were located in proximity to each other forming the active site, as expected. We next analyzed the locations of PTRPLS and PTRPLS-like mutations in our model (Fig. 6). PTRPLS mutants D349N, G393E, and G394E are located in the putative active site. Y366 is located between the ^349^DDD^351^ and ^393^GGG^395^ motifs. Nonsense mutation at Y366 eliminated all downstream residues, including the G-loop (Figs. 6 and S7A). Although PTRPLS R412∗ also resulted in elimination of downstream residues, it retained both the ^349^DDD^351^ and ^393^GGG^395^ motifs (Figs. 6 and S7B). None of the PTLRPLS-like mutations are located near the C-GT active site.

**Figure 7:**
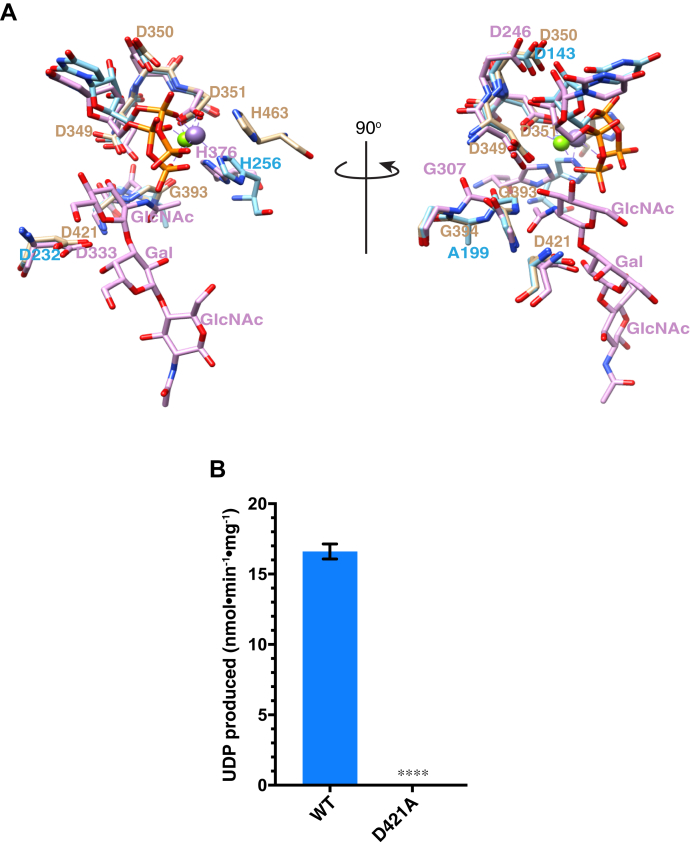
**Structure alignment of B3GLCT C-GT with other GT31 family proteins identified D421 as the catalytic base.***A*, the UDP ligands for MFNG (PDB 2J0B, *blue*) and B3GNT2 (PDB 7JHN, *purple*) are displayed along with catalytic base, D232 for MFNG and D333 for B3GNT2. D421 of B3GLCT overlayed with D232 in MFNG and D333 in B3GNT2. DxD motifs and G-loops of all three enzymes also overlayed. *B*, *in vitro* enzyme assay (20 min) of B3GLCT WT and D421A mutant with UDP-Glc and Fuc-*O*-TSR3. Statistical analysis was performed with paired *t* test, two-tailed in Prism 7, n = 3, ∗∗∗*p* < 0.001.

To validate our B3GLCT C-GT model, we aligned the C-GT model with structures of MFNG (PDB 2J0B) and B3GNT2 (Fig. S8). The backbone of B3GLCT C-GT overlayed with MFNG and B3GNT2 with an overall RMSD (root-mean-square deviations of atomic positions) of 1.7 Å (Fig. S8). Amino acid sequence alignment between these three proteins revealed that the DxD motifs, G-loops, and xED motifs are highly conserved among all three proteins (Fig. S9). This conservation was reflected locally in structural alignment as well as the DxD motif, G-loop, and xED motif of B3GLCT C-GT also superimposed with those in MFNG and B3GNT2 (Fig. 7*A*). The C-terminal His in MFNG, B3GNT2, and B3GLCT C-GT also aligned in the structure (Fig. 7*A*), but not in the primary sequence (Fig. S9), likely due to the fact that it occurs in the hypervariable region of GT-A fold enzymes with significant sequence variability (6). Because the UDPs in MFNG and B3GNT2 overlayed with each other (Fig. 7*A*), we predict that the ^349^DDD^351^ in B3GLCT interacts with UDP-Glc in a manner similar to how MFNG and B3GNT2 interact with UDP-GlcNAc. With acceptor substrate (GlcNAc-1,3-Gal-1,4-GlcNAc) *in loco* in the B3GNT2 structure, we identified D421 in B3GLCT as the catalytic base based on the overlay of D421 with catalytic bases D232 in MFNG and D333 in B3GNT2 (Fig. 7*A*). We then generated a D421A mutant to assess its impact on catalytic activity. As expected, the D421A mutant completely lost the ability to transfer Glc to Fuc-*O*-TSR3 compared with WT B3GLCT (Fig. 7*B*). This data strongly supports our prediction that D421 is the catalytic base of in B3GLCT and also validated our structural model of B3GLCT C-GT.

MFNG utilizes Mn^2+^ as a cofactor (9) while B3GNT2 utilizes Mg^2+^ (10, 11) (Fig. 7*A*, left panel). However, high concentration of EDTA did not abolish the catalytic activity of B3GLCT (Fig. S10). Low concentrations of MgCl_2_ and MnCl_2_ slightly increased catalytic activity, while higher concentrations of MnCl_2_ and CaCl_2_ inhibited the reaction (Fig. S10). These data implied that B3GLCT may not require a metal ion for catalyzing the transfer of Glc to *O*-fucosylated TSRs.

To further analyze the structure of the N-GT-like domain, we compared the B3GLCT N-GT-like domain homology model with the B3GLCT C-GT model, MFNG structure, and B3GNT2 structure (Fig. S11*A*). The N-GT-like domain also adopts a GT-A fold as predicted by sequence analysis, and the putative DxD motif and G-loop are close to each other, suggesting a potential active site (Fig. S11*B*). A conserved C-His is missing. Based on the model alignment, we identified D216 (Fig. S11*B*) from B3GLCT N-GT-like domain as the putative catalytic base; however, D212 was identified as putative catalytic base during amino acid sequence analysis (Figs. 1*B* and S1). Hence, we were not able to conclusively predict a putative catalytic base for the N-GT-like domain or whether it is likely to have glycosyltransferase activity.

## Discussion

Although both PTRPLS and PTRPLS-like patients have mutations in *B3GLCT*, our data demonstrate that the PTRPLS mutations eliminate B3GLCT function while the PTRPLS-like mutations do not. PTRPLS mutations ablate *in vitro* B3GLCT enzymatic activity and the ability to rescue secretion of ADATMS20 TSR2-8 from *B3GLCT*^−/−^ cells. Together these results demonstrate that the molecular mechanism for the phenotypes observed in PTRPLS patients is loss of glucose on B3GLCT substrate proteins and not an unknown function of the N-GT-like domain. These results also confirm that loss of secretion of B3GLCT substrates such as ADAMTS20 from *B3GLCT*^*−/−*^ cells is due to loss of glucose and not to the chaperone effect of an enzymatically inactive enzyme.

We demonstrated that the C-GT domain, not the N-GT-like domain, is solely responsible for catalyzing the transfer of Glc to *O*-fucosylated TSRs, using a catalytic base at D421 (Figs. 2 and 7). However, B3GLCT appears to require the presence of both domains to maintain the overall structure and function since neither the N-GT-like nor C-GT domains could be expressed on their own (Fig. S3, *B* and *C*). The function of the N-GT-like domain is unknown. Righino *et al.* (32) modeled two GT-A fold domains in LARGE1, but the two domains are not closely related to one another and catalyze distinct reactions. In the case of B3GLCT, sequences between the two GT domains are more closely related to each other than to any other proteins within the GT31 family (8). The C-GT domain has all of the conserved features of the GT-A fold glycosyltransferase family, including the conserved DxD motif, G-loop, xED motif, and C-His (6). While glycosyltransferases with the DxD motif typically require a divalent cation for activity, our data demonstrated that B3GLCT was functional even in the presence of EDTA (Fig. S10). It is possible that a divalent ion is bound very tightly and not accessible to removal by EDTA. Sequence analysis (Fig. 1*B*) and homology modeling (Fig. S11) of the N-GT-like domain reveal the presence of a putative DxD motif, G-loop, and catalytic base, suggesting it may be a glycosyltransferase, but confirmation will require demonstration of enzymatic activity. Alternatively, the N-GT-like domain could play a role in recognizing substrates, possibly binding an adjacent TSR since most substrates contain multiple, tandem TSRs. It may play a role in the cooperative binding of *O*-fucosylated TSRs observed in Figure 3*B*. Since expression of B3GLCT is dependent on the presence of both the N-terminal and C-terminal domains (Fig. S3), another intriguing possibility is that the N-GT-like domain serves as a chaperone for the folding of the C-GT domain, similar to the way that COSMC functions as a chaperone for the folding of core 1 β3-galactosyltransferase (33). Further study with structural characterization of B3GLCT should help to better understand the role of the N-GT-like domain and whether metal ions are needed for catalysis.

The PTRPLS mutations evaluated here had a variety of effects on B3GLCT activity and function. The most dramatic effects were those in the conserved DxD motif, D349N, and G-loop, G393E. Both of these mutations completely inhibited transfer of glucose to Fuc-*O*-TSR3, and neither rescued secretion of ADAMTS20 TSR2-8 from *B3GLCT*^−/−^ cells. Based on our sequence analysis and comparison with MFNG and B3GNT2, we predict that D349 in ^349^DDD^351^ and G393 in ^393^GGG^395^ behave and function in the same fashion as D245 and G306 in B3GNT2. In the cocrystal structure of B3GNT2 with UDP and acceptor GlcNAcGalGlcNAc, both D245 in the DxD motif and G306 in the G-loop of B3GNT2 interact with the donor substrate UDP-GlcNAc, and the structural shift of G307 together with the whole ^304^GGGG^307^ loop also stabilizes B3GNT2 upon acceptor substrate binding (11). Hence, the loss of function caused by D349N is likely due to loss of the interaction with UDP-Glc, while G393E disrupts the flexibility and shift of the G-loop (Figs. 6 and 7*A*, right panel).

Other PTRPLS mutations that could not be expressed in sufficient quantity for *in vitro* assays appeared to have a significant effect on B3GLCT stability: G394E, Y366∗, and R412∗. G394E and Y366∗ did not rescue the secretion of ADAMTS20 TSR2-8 in *B3GLCT*^−/−^ cells, while R412∗ did so poorly. All of these PTRPLS mutations were expressed at low levels in cells, suggesting that the mutations cause destabilization of the protein resulting in degradation by the ER-associated degradation (ERAD) pathway (34). Interestingly, the G393E mutant reduced melting temperature and also slightly reduced B3GLCT cellular levels. We saw no evidence that any PTRPLS mutants, including the nonsense mutants, affect the localization of B3GLCT as we did not detect any B3GLCT secreted to the media in any of our cell-based rescue assays.

In addition to affecting stability, the nonsense mutations Y366∗ and R412∗ removed important catalytic residues that should eliminate enzymatic activity. The Y366∗ mutant removed both the G-loop (^393^GGG^395^) and the catalytic base D421 (Fig. S5*A*), affecting both substrate binding and catalysis. The R412∗ mutant has both DxD motif and the G-loop to bind UDP-Glc but is unlikely to catalyze the transfer reaction due to loss of the catalytic base, D421 (Fig. S5*B*). It was surprising that R412∗ could even rescue ADAMTS20 TSR2-8 secretion partially, especially because other enzymatically inactive mutants (*i.e.*, D349N and G393E) could not (Fig. 4). This could possibly be due to a residual ability of the R412∗ mutant to bind and stabilize folded TSRs, slightly enhancing secretion. Alternatively, the R412∗ mutant could have low enzymatic activity, although we cannot produce sufficient amounts of the protein for an *in vitro* assay. The reduced protein levels of both Y366∗ and R412∗ are likely caused by deletion of large portions of B3GLCT resulting in misfolding and rapid degradation of truncated protein.

Although PTRPLS-like mutants retained enzymatic activity and the ability to rescue secretion of ADAMTS20 TSR2-8, several of these mutations reduced the stability (Q457R, V245M, R337H), and all had a small effect on the enzyme turnover numbers of B3GLCT. The reduced turnover numbers may be due to the destabilization of these mutants during the *in vitro* assays at 37 °C. Nonetheless, these mutations do not appear to be severe enough to cause loss of secretion of B3GLCT substrate proteins from cells. Since these mutations in *B3GLCT* have been reported as heterozygous in PTRPLS-like patients, along with the fact that most PTRPLS-like patients have no mutations in *B3GLCT* (3), these mutations are more likely polymorphisms with little effect on the biological function of B3GLCT.

Together, our data suggests that PTRPLS mutations affect the function and stability of B3GLCT, resulting in loss of glucose and reduced secretion of substrate proteins, such as ADAMTS9 (26), ADAMTS17 (25), and ADAMTS20 (26), that play important roles in development processes of the eyes, bones, brain, and other systems, resulting in PTRPLS phenotypes. There are other B3GLCT substrate proteins involved in bone development where secretion is not affected by loss of B3GLCT, such as ADAMTSL2 (27, 30). Thus, PTRPLS is likely caused by reduced secretion and function of some but not all B3GLCT substrates. Further studies need to be done on additional B3GLCT substrate proteins to fully understand the molecular mechanisms of the phenotypes observed in PTRPLS patients. Better understanding of the disease mechanism is essential to advance the development of therapeutics for PTRPLS patients.

## Experimental procedures

### Domain annotation and sequence alignment

The domain map of human B3GLCT was annotated using a profile-based search from sequence profiles built from a previous study (6). This search generated an alignment to existing GT31 sequences, allowing the identification of each domain. The N-terminal DxD motif was identified through this alignment. These domains were further refined through searches using the Conserved Domain Database (35), confirming the absence of the G-loop, catalytic base motif, and the C-terminal Histidine in the N-GT-like domain. Multiple sequence alignment (Figs. 1*B* and S1) was performed *via* the MAFFT program (36). Sequence comparison in Figure S9 was performed with Clustal Omega program through EMBL-EBI website server (37).

### Plasmids and site-directed mutagenesis

pcDNA3.1(+)-hB3GLCT encoding full-length, untagged human B3GLCT has been described before (5) and was generously provided by Dr Hisashi Narimatsu (NIAST, Japan). His_6_-B3GLCT-FL (pcDNA3.1(+)-His_6_-hB3GLCT) for cell-based secretion assays evaluating nonsense mutations was generated by PCR using CloneAmp HiFi PCR Premix (Takara Bio Inc.) with primers 5’- CATCATCATCATCATCATGAGGTCAAGCAGTCTCAGG-3’ and 5’-TTTCTTTGTATCTTCAGAAGCC-3’. After PCR, the fragments were treated with T4 Polynucleotide Kinase and T4 Polynucleotide Ligase (NEB) to make circular plasmid. For protein expression and purification, we generated B3GLCTΔREEL (pSecTag2/HygroC-hB3GLCTΔREEL-Myc-His_6_) by amplifying the open reading frame (ORF) for the hB3GLCTΔREEL fragment with primers: 5’-ATATATGGTACCTATCTGAAGATACAAAGAAAGAGGTCAA-3’ and 5’-ATCTAGCTCGAGCAAAACCTTTCTGTGTCTCCTGC-3’. They include KpnI or XhoI restriction enzyme site for subcloning, respectively, into pSecTag2/HygroC. All mutations were introduced to pcDNA3.1(+)-B3GLCT (for cell-based assays), pcDNA3.1(+)-His_6_-B3GLCT (for cell-based assays of nonsense mutations) and pSecTag2/HygroC-hB3GLCTΔREEL-Myc-His_6_ (for protein expression) by site-directed mutagenesis using primers in Table S1 and CloneAmp HiFi PCR premix (Takara Bio Inc.) with manufacture recommended conditions. Three kinds of hB3GLCT C-GT domain construct were generated by using PCR with CloneAmp HiFi PCR Premix (Takara Bio Inc.). We used pcDNA3.1(+)-hB3GLCT (for cell-based assays) and pSecTag2/HygroC-hB3GLCTΔREEL-Myc-His_6_ (for protein expression) as template. After PCR, the fragments were treated with T4 Polynucleotide Kinase and T4 Polynucleotide Ligase (NEB) to make circular plasmid. Three kinds of hB3GLCT N-GT-like domain constructs were generated using pcDNA3.1(+)-His_6_-B3GLCT by introducing stop codon at C241, T252, and K262. PCR reactions used CloneAmp HiFi PCR Premix (Takara Bio Inc.). Primers that we used are listed in Table S1. pSecTag2-mAdamts20TSR2-8-Myc (for cell-based assay evaluating truncation mutants) was produced by inserting stop codon with reverse PCR at the first histidine of His_6_-tag in pSecTag2-mAdamts20TSR2-8-Myc-His_6_ (26) with forward primer (5’-3’ CGTCGACTAGCATCATCATCATCATTGAGT) and reverse primer (5’-3’ GATGATGCTAGTCGACGGCGCTATTCA). The PCR reaction was performed with CloneAmp HiFi PCR premix (Takara Bio Inc.). All plasmids were confirmed by sequencing.

### Protein expression and purification

HEK293T (ATCC) was cultured and seeded in Dulbecco’s Modified Eagle Medium (DMEM, GE Healthcare Life Sciences) supplemented with 10% bovine calf serum (BCS, VWR) and 1% penicillin plus streptomycin (pen + strep, Sigma-Aldrich). Before transfection, cells were washed once with Dulbecco’s phosphate buffered saline (Sigma-Aldrich) and 6 ml Opti-MEM (Gibco) was added for each 10 cm culture dish. The cells were transiently transfected with pSecTag2/HygroC-hB3GLCTΔREEL-Myc-His_6_ WT or PTRPLS/PTRPLS-like mutants using 5 μg plasmid, 30 μL of 1 mg/ml polyethylenimine (PEI, (38)), and 500 μL Opti-MEM. The plasmid, PEI, and Opti-MEM were incubated at room temperature for 15 min before addition to cells. After 3-day incubation, culture medium was collected and centrifuged at 3900 rpm at 4 °C for 20 min. The supernatant was filtered through 0.45 μm filter and purified using Ni-NTA (Qiagen) affinity chromatography at 4 °C. The proteins were eluted in Tris buffered saline pH 7.5 (TBS) containing 250 mM imidazole. The eluents were buffer exchanged to 10% glycerol in TBS with 10 kDa molecular weight cutoff centrifugal filters (Amicon) with manufacturer recommended conditions at 4 °C and stored at –80 °C until use. Concentrations of purified proteins were measured by absorbance at 280 nm using extinction coefficient 87,320 with NanoDrop (Thermo Fisher Scientific). The extinction coefficient was calculated using human B3GLCT fasta sequence (Uniprot Q6Y288) on ProtParam tool in ExPASy (39). Purity of collected proteins was analyzed by SDS-PAGE and Coomassie blue staining.

### *In vitro* enzyme assays

All protein activity and enzyme kinetic assays were performed with *UDP Glo Glycosyltransferase Assay* from Promega Corporation. Each reaction contained 0.5 μg purified enzyme, 50 μM Ultra-pure UDP-Glc (Promega), and 25 μM Fuc-*O*-TSR3 (or storage buffer containing 0.25 mM β-mercaptoethanol in 100 mM Tris/HCl pH 8.0 for background control) in 50 mM Tris/HCl pH 7.5 in 20 μL final reaction volume. Reactions were incubated at 37 °C for 20 min and were stopped by putting samples on ice. In the metal ion screening experiment, each metal ion/EDTA was added from 10× stock solution using H_2_O to adjust the final volume to 20 μL. The time-dependent experiment was performed with 4-nitrophenl-α-L-fucopyranoside (pNP-Fuc, Sigma-Aldrich) as acceptor substrate dissolved in DMSO (Fisher Scientific). All reactions used 10× pNP-Fuc stocks to have an equal percentage of DMSO in each reaction. After the transfer reaction, all samples were incubated with nucleotide detection reaction (NDR, Promega) and buffer diluted Glo Enzyme (Promega) to detect produced UDP from reaction using luminescence for detection. Produced UDP after each reaction was quantified based on UDP standards (Promega) and converted to desired units. All reactions were performed in biological triplicates with three batches of purified enzymes. Fuc-*O*-TSR3 kinetics were analyzed with nonlinear Allosteric sigmoidal least squares (ordinary) fitting in Prism 7. UDP-Glc kinetics were calculated with nonlinear Michaelis–Menten least squares (ordinary) fitting in Prism 7.

### Bacterial protein expression and purification

Unmodified human thrombospondin-1 TSR3 (pET21b(+)-hTSP1-TSR3) was expressed from *Escherichia coli* BL21 strain (Invitrogen) and purified *via* reverse-phase high-performance liquid chromatography (HPLC, Agilent) as described (14). Fuc-*O*-TSR3 was produced by modifying human thrombospondin-1 TSR3 in *in vitro* reactions containing 20 μM TSR3, 3 ng/μL purified POFUT2, 100 μM GDP-fucose, and 1 mM MnCl_2_ in 50 mM HEPES pH 6.8 as described (40). The reaction was incubated at 37 °C overnight. Fuc-*O*-TSR3 was purified from the reaction mixture using HPLC and confirmed with mass spectrometry.

### Cell-based secretion assays

CRISPR-Cas9 HEK293T knockout of *B3GLCT* was generated as previously described (25). Cells were seeded in 2 ml (4.24 × 10^6^ cells/ml) DMEM supplemented with 10% BCS in 6-well plates, incubated overnight, and transfected when 80% confluent. Transient cotransfection of WT or *B3GLCT*^−/−^ cells was performed with 1.66 μg ADAMTS20 TSR2-8 plasmid, 0.1 μg GFP plasmid (pEGFP-N1, Clontech) as a transfection and protein loading control, 0.24 μg of pcDNA3.1(+)-hB3GLCT or pcDNA3.1(+)-His_6_-B3GLCT WT or mutant for rescue experiments (pcDNA3.1(+) empty vector as a negative control for compensate total transfected plasmid amount) with 12 μL PEI in 200 μL Opti-MEM incubated at room temperature for 15 min. Before transfection, medium was changed to 700 μL Opti-MEM and the transfection mixture was added drop-wise. Cells were then cultured for 48 h before collecting medium and cells for western blot analysis. Serial dilution of the *B3GLCT* plasmids was performed starting from 0.24 μg as the highest, 0.048 μg as 5-fold reduce, 0.024 μg as 10-fold reduced, and 12 ng as 20-fold reduced. The total cotransfection plasmid amount of serial diluted plasmids was maintained by addition of appropriate amount with pcDNA3.1(+) empty vector. All cell-based assays were performed with biological triplicates with representative figures chosen for demonstration.

### Western blot

For secreted protein detection, 50 μL of collected media was precipitated with 200 μL ice-cold acetone (Fisher Scientific) at –20 °C overnight. The precipitated protein pellet was harvested by 127,000 rpm centrifuge (Eppendorf) at 4 °C for 10 min and resuspended with 20 μL 2× denaturing sample buffer (Tris/HCl 100 mM pH6.8, glycerol 20%, sodium dodecyl sulfate (SDS) 0.04% w/v, bromophenol blue 0.2% w/v, 2-mercaptoethanol 200 mM) by sonication for 15 min and boiling at 100 °C for 5 min. Cell lysate was collected by lysing transfected cells with 200 μL 1% Nonidet P-40 (NP-40, US Biological Life Sciences) in TBS and centrifuged at 110,000 rpm (Eppendorf) 4 °C 15 min 15 μL of cell lysate was used for analysis. Samples were resolved on 4–20% gradient tris-glycine gels (Invitrogen), and proteins were transferred to 0.45 μm nitrocellulose membrane (Bio-Rad). Membranes were blocked at 4 °C overnight in 5% nonfat milk (Bio-Rad) in TBS with 0.1% Tween 20, then probed with anti-Myc antibody (9E10, Stony Brook University Cell Culture/Hybridoma facility), anti-GFP (Cell Signaling Technology, cat. no. 2555), and anti-B3GLCT (OriGene, cat. no. TA316142) with 1:2000 dilution for each antibody at room temperature for 2 h. Membranes were then incubated with Alexa Fluor IRDye680RD goat anti-mouse (LI-COR, cat. no. 926-68070) and IRDye800CW goat anti-rabbit (LI-COR, cat. no. 925-32211) with 1:10,000 dilution at room temperature for 1 h. N-His_6_-B3GLCT and ADAMTS20 TSR2-8-Myc were detected with anti-His-tag (Bio-Rad, cat. no. MCA1396) and anti-Myc (Thermo Fisher Scientific, cat. no. PA1-981) with same dilutions and incubation conditions as described above. Antibody to detect GFP in the attempted expression of B3GLCT C-GT domains was chicken anti-GFP (Abcam, ab13970) with donkey anti-chicken IRDye680RD (LI-COR, 925-68075) as secondary antibody. Immunoblots were visualized on Odyssey Imager (LI-COR) software.

### Protein stability assays

Protein stability was analyzed with thermo shift assays by measuring the fluorescence of SYPRO Orange (Thermo Fisher Scientific) upon binding the hydrophobic pocket of proteins with increased temperature gradient with manufacturer recommended protocol (Bio-Rad CFX Real Time PCR Detection System, Protein Thermo Shift). Briefly, purified protein (0.1 μg) was mixed with 5× SYPRO Orange using 10% glycerol in TBS to adjust final volume to 25 μL. Temperature gradient was applied as 15–95 °C with 0.5 °C ramp on Bio-Rad CFX96 real-time qPCR machine. Melting temperature was determined by the temperature (X-axis) at the peak of melting peak from CFX Maestro Software (Bio-Rad). Melting peaks were generated as the first derivatives of the melting curve by the CFX Maestro Software (Bio-Rad) during the run. Experiment was repeated with two batches of purified enzymes with six replicates in total. Melting temperatures of WT and mutants were analyzed and graphed in Prism 7. Statistical analysis was performed with One-way ANOVA in Prism 7.

### Homology modeling

B3GLCT homologous model was generated using the SWISS MODEL website server with ProMod3 version 3.0.0 (41, 42, 43, 44, 45). Full-length B3GLCT (UNIPROT Q6Y288) was used as subject sequence. Homology model of B3GLCT C-GT domain and N-GT-like domain were generated based on mouse MFNG crystal structure (PDB 2J0A) with B3GLCT sequence coverage 40.6% and 37.4%, respectively. All protein structures were visualized and analyzed in Chimera (46).

## Data availability

All original images of immunoblotting and homology modeling details are available upon request.

## Supporting information

This article contains supporting information.

## Conflict of interest

The authors declare that they have no conflicts of interest with the contents of this article.
