# Supplementary material for: Peters plus syndrome mutations affect the function and stability of human β1,3-glucosyltransferase
Source: J Biol Chem. 2021 May 28;297(1):100843. doi: 10.1016/j.jbc.2021.100843 (PMC8233153; doi:10.1016/j.jbc.2021.100843)
Supplement: Figures S1–S11 and Table S1 [file mmc1.pdf]

Supporting Information for:

Peters plus syndrome mutations affect the function and stability of human β1,3-glucosyltransferase

Ao Zhang, Aarya Venkat, Rahil Taujale, James L. Mull, Atsuko Ito, Natarajan Kannan, Robert S. Haltiwanger

Contents:

Table S1

Figures S1-S11

Supporting Information Table:

Table S1. Site-directed mutagenesis primers for human B3GLCT construct.

Clone	Primer name	Sequence (5'→ 3')
D349N	AI73 f	AGTCATTGTGAATGATGATACATTAATAAGTATCTCCAGGCTCC
	AI74 r	GTATCATCATTCACAATGACTAACCATGCTGTTTTGTCCTG
G393E	KZ21 f	CACGGGAGAAGGAGGAATGGTCTTCAGCAGAG
	KZ22 r	ATTCCTCCTTCTCCCGTGATGTAGCTGTAGCC
G394E	KZ23 f	GGGAGGAGAAGGAATGGTCTTCAGCAGAGAAG
	KZ24 r	ACCATTCCTTCTCCTCCCGTGATGTAGCTGT
Q457R	KZ07 f	TTCTCATCGGGTTCCCATATCGTTCCACAAAC
	KZ08 r	ATGGGAACCCGATGAGAAAGGTAGTCCTTAGG
T179S	KZ41 f	CGAGAATCCTTCAGTTTTTAAGTATCCAGACTTTG
	KZ42 r	CTTAAAAACTGAAGGATTCTCGGAAAAGGCATAATG
V245M	KZ43 f	GTACCAATGACATGGACTTCTACTGTGCTACCACATTC
	KZ44 r	GTAGAAGTCCATGTCATTGGTACAAAACTCAGGCACTG
R337H	KZ45 f	TTTCTGAATCATAGCCAGGACAAAACAGCATGG
	KZ46 r	GTCCTGGCTATGATTCAGAAATCTTTCCAAAATGG
Y366*	KZ17 f	AGCTGTTAAGACTCCGGCGAGCCTGTGTTT
	KZ18 r	GCCGGAGTCTTAACAGCTAAGCAAGTGCTG
R412*	KZ19 f	AGTAAATGTTGATGCTACAGCAATGATGCTC
	KZ20 r	CTGTAGCATCAACATTTACTGGCGAGAA
E132A	KZ49 f	CTTCTGTGCCGAAGAGACAAGAATACAGATTCCAAAACTCTTGG
	KZ50 r	CTTGTCTCTTCGGCACAGAAGAAAATCCAAGATGAATTTCTGCTA
D421A	KZ55 f	TCCCGATGCCATGGTCCTGGGAATGTGCTTTAG
	KZ56 r	AGGACCATGGCATCGGGAGCATCATTGCTGTAGC
CT1	AI210 f	GAGTTTTGTACCAATGACGTGGACTTCTAC
	AI214 r (pcDNA3.1)<br>AI213 r (pSecTag2C)	AGCCAAACCAAAAGCCAGGGAG<br>GGCCGCGTCACCAGTGG
CT2	AI211 f	ACATTCCATTCTTTTCTACCGCTTTGTAG
	AI214 r (pcDNA3.1)<br>AI213 r (pSecTag2C)	AGCCAAACCAAAAGCCAGGGAG<br>GGCCGCGTCACCAGTGG

CT3	AI212 f	AAGCCAGTGAAGAAGAAGGATATTTTTGTTG
	AI214 r (pcDNA3.1)<br>AI213 r (pSecTag2C)	AGCCAAACCAAAAGCCAGGGAG<br>GGCCGCGTCACCAGTGG
NT1	AI337 f	CTGAGTTTTGAACCAATGACGTGGACTTCTACTGTG
	AI338 r	CGTCATTGGTTCAAAACTCAGGCACTGGGGTC
NT2	AI339 f	TGTGCTACCTGATTCCATTCTTTTCTACCGCTTTGTAGAAAGCC
	AI340 r	GAATGGAATCAGGTAGCACAGTAGAAGTCCACGTCATTGG
NT3	AI341 f	GCTTTGTAGATAGCCAGTGAAGAAGAAGGATATTTTTGTTGC
	AI342 r	TTCACTGGCTATCTACAAAGCGGTAGAAAAGAATGGAATG

Supporting Information Figure Legends:

Supporting information

Figure S1. Alignment of B3GLCT sequences from multiple species. Multiple sequence alignment of B3GLCT from human (UNIPROT Q6Y288), mouse (UNIRPOT Q8BHT6), zebrafish (UNIRPOTA0A068F9P7), fruitfly (UNIPROT X2JDC2), and starfish (NCBI REFSEQ XP_038056415.1) were aligned with the MAFFT software/algorithm. Red background highlights regions of 100% sequence identity within the shown sequences; red letters indicate regions of 100% sequence similarity; blue rectangles enclose sets of conserved residues. Annotated domains are highlighted under amino acid sequences.

Figure S2. Time-dependent *in vitro* enzyme assay to determine the linear phase of the transfer reaction catalyzed by B3GLCT. 0.5 μg purified B3GLCT, 50 μM UDP-Glc and three different concentrations of pNP-Fuc were analyzed. Reactions were stopped by freezing on dry ice. All reactions were incubated at 37 °C.

Figure S3. Neither the B3GLCT C-GT nor N-GT-like domains expressed alone. (A) Domain map of human B3GLCT with amino acids cutoff for N-GT-like domain and C-GT domain constructs. Each N-GT-like domain (NT1, NT2, NT3) or C-GT domain construct (CT1, CT2, CT3) contained amino acids ending or beginning as indicated. SP, signal peptide; N-GT-like, N-terminal GT-like domain; C-GT, C-terminal GT domain; REEL, C-terminus containing KDEL-like motif. **(B)** Plasmids encoding ADAMTS20 TSR2-8-Myc-His_6 and GFP were co-transfected into WT or *B3GLCT*$^{-/-}$ HEK293T cells. Rescue experiments were performed by co-transfection

with plasmids encoding full-length WT B3GLCT (wtB3), or the CT1, CT2 or CT3 constructs, all with the REEL ER-retention motif. Media and cell lysates were analyzed by Western blot probed with anti-Myc (red, 50 kDa) to detect ADAMTS20 TSR2-8 (TSR2-8), and anti-B3GLCT for B3GLCT WT or mutants (green, 50 kDa, mutants expected <50kDa). Anti-GFP for transfection and loading control (red, 25 kDa). **(C)** Plasmids encoding WT, NT1, NT2, and NT3, all generated with the N-His6-B3GLCT construct, were co-transfected into WT HEK293 cells with GFP plasmid. Media and cell lysates were analyzed by Western blot probed with anti-His_6-tag (red, WT 50 kDa, mutants <50 kDa) and anti-GFP for transfection (green, 25 kDa).

Figure S4. Coomassie blue staining of purified WT and PTRPLS/PTRPLS-like mutant enzymes. Bovine serum albumin (BSA) standards and purified B3GLCT WT, PTRPLS (black) and PTRPLS-like (blue) mutants were separated by 10% SDS-PAGE and stained with Coomassie blue for imaging. For purified enzymes, 5 μL of eluate was loaded in each lane.

Figure S5. B3GLCT with PTRPLS mutations did not rescue secretion of ADAMTS20 TSR2-8 while PTRPLS-like mutations did. Plasmids encoding ADAMTS20 TSR2-8-Myc-His_6 (TSR2-8) and GFP were co-transfected into wild type (WT) and *B3GLCT* knockout (*$B3GLCT^{-/-}$)* HEK293T cells. PTRPLS mutations are colored in black and PTRPLS-like mutations in blue. Rescue experiments were performed by co-transfection with a plasmid encoding full-length B3GLCT WT, G393E, and G394E **(A)**, Q457R and T179S **(B)**, and V245M and R337H **(C)** (B3GLCT) with serial dilutions. Serial dilutions of B3GLCT plasmids were performed starting with 0.24 μg of plasmid diluted 5-, 10-, and 20-fold. Media and cell lysates were analyzed by Western blot probed with anti-Myc (red) to detect ADAMTS20 TSR2-8, anti-

B3GLCT to detect endogenous or transfected B3GLCT (green, 50 kDa), and anti-GFP for transfection and loading control (green, 25 kDa).

Figure S6. B3GLCT mutants G393E and Q457R destabilized the enzyme. Melting curve **(A)** and first derivative showing the melting peak **(B)** of WT and mutants were analyzed using the thermo shift protein stability assays described in Experimental Procedures. 10% glycerol in TBS was used as negative control to monitor the background. Arrows in **(B)** point to reduced melting temperature of G393E (blue) and Q457R (orange).

Figure S7. PTRPLS nonsense mutations at Y366 and R412 result in deletion of important catalytic residues. Ribbon presentation of structures for B3GLCT C-GT Y366* **(A)** and R412* **(B)**. Light blue ribbon represented the backbone of B3GLCT C-GT. Light blue, residues without mutations; salmon, residues at which had PTRPLS mutations; yellow, residues at which had PTRPLS-like mutations (blue letters).

Figure S8. Internal core structures of B3GLCT C-GT aligned with the cores of MFNG and B3GNT2. Protein backbone alignment between B3GLCT C-GT model (tan), MFNG (PDB 2J0B, blue) and B3GNT2 (PDB 7JHN, purple). Structure alignment was visualized with Chimera.

Figure S9. Sequence alignment revealed that B3GLCT C-GT has conserved motifs of GT31 family enzymes. Amino acid sequence of human B3GLCT (UNIPROT Q6Y288) was aligned to

the sequences of human B3GNT2 (UNIPROT Q9NY97) and mouse MFNG (UNIPROT O09008). DxD motif, dark blue; Glycine rich loop, magenta; xED motif, purple; C-His, orange.

Figure S10. B3GLCT is active in the absence of divalent cations. *In vitro* enzyme assays of B3GLCT with UDP-Glc and Fuc-*O*-TSR3 were supplied with various divalent metal ions at three different concentrations. 10x stock solutions of $MnCl_2$, $MgCl_2$, $CaCl_2$ and EDTA were supplemented to each reaction to make final in-reaction concentrations as described. Statistical analysis was performed with One-way ANOVA in Prism 7. Error bars, standard deviation, n=3; $^{*}p<0.05$; $^{**}p<0.01$.

Figure S11. B3GLCT N-GT-like domain adopts a GT-A fold. (A) Protein backbone alignment between B3GLCT N-GT-like domain (tan), B3GLCT C-GT model (green), MFNG (PDB 2J0B, blue) and B3GNT2 (PDB 7JHN, purple). Structure alignment was visualized with Chimera. **(B)** The DxD motif, G-loop, catalytic aspartic acid, and C-His for B3GLCT N-GT-like domain model (tan), C-GT domain model (green) are aligned with MFNG (PDB 2J0B, blue), B3GNT2 (PDB 7JHN, purple). DxD motifs, G-loops, and catalytic aspartic acid of all four domains also overlayed, whereas C-His of the N-GT domain is missing.

Figure S1

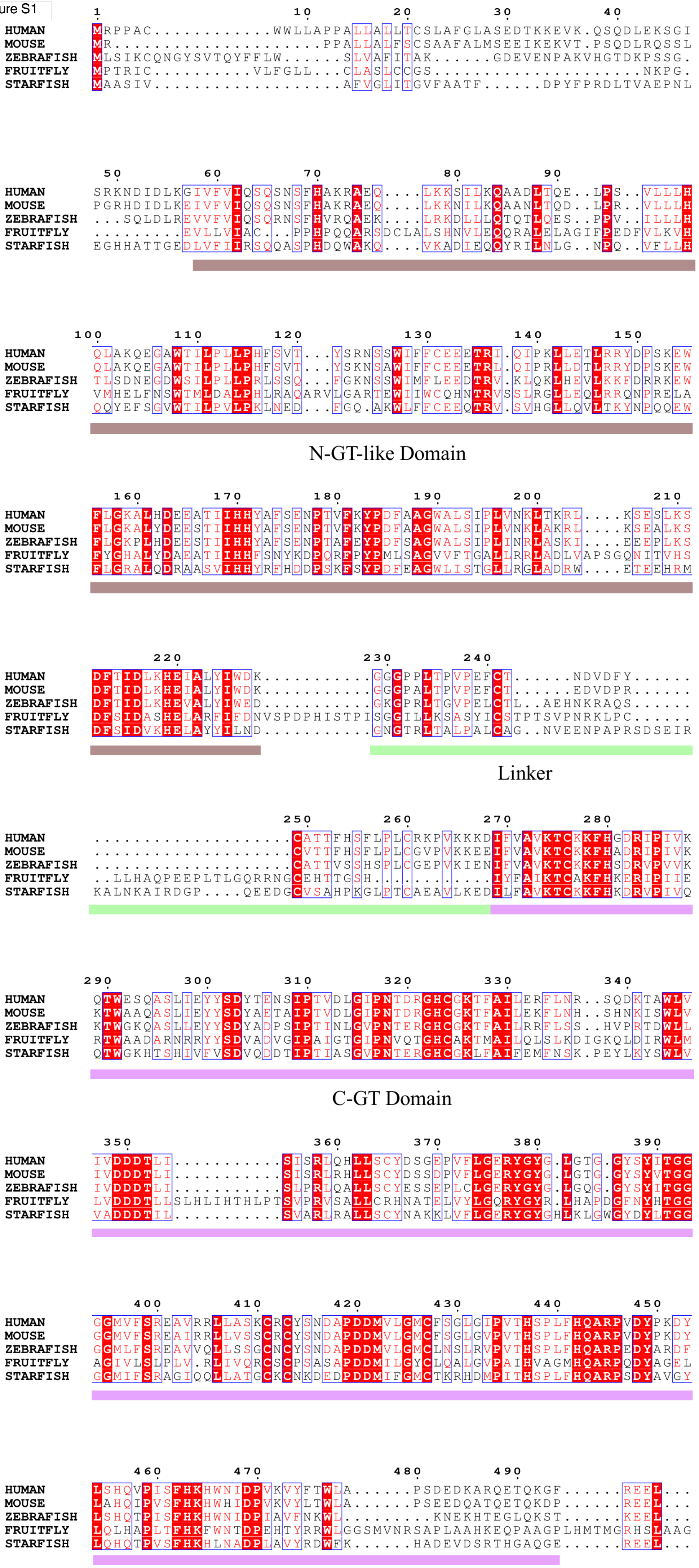

1 10 20 30 40
HUMAN MRPPAC............WWLLAPPALLALLTCSLAFGLASEDTKKEVK.QSQDLEKSGI
MOUSE MR..................PPALLALFSCSAAFALMSEEIKEKVT.PSQDLRQSSL
ZEBRAFISH MLSIKCQNGYSVTQYFFLW......SLVAFITAK......GDEVENPAKVHGTDKPSSG.
FRUITFLY MPTRIC.........VLFGLL...CLASLCCGS...................NKPG.
STARFISH MAASIV..................AFVGLITGVFAATF......DPYFPRDLTVAEPNL
50 60 70 80 90
HUMAN SRKNDIDLKGIVFVIQSQSNSFHAKRAEQ....LKKSILKQAADLTQE..LPS..VLLLH
MOUSE PGRHDIDLKEIVFVIQSQSNSFHAKRAEQ....LKKNILKQAANLTQD..LPR..VLLLH
ZEBRAFISH ...SQLDLREVVFVIQSQRNSFHVRQAEK....LRKDLLLQTQTLQES..PPV..ILLLH
FRUITFLY ....EVLLVIAC...PPHPQQARSDCLALSHNVLEQQRALELAGIFPEDFVLKVH
STARFISH EGHHATTGEDLVFIIRSQQASPHDQWAKQ....VKADIEQQYRILNLG..NPQ..VFLLH
100 110 120 130 140 150
HUMAN QLAKQEGAWTILPLLPHFSVT...YSRNSSWIFFCEEETRI.QIPKLLETLRRYDPSKEW
MOUSE QLAKQEGAWTILPLLPHFSVT...YSKNSAWIFFCEEETRL.QIPRLLDTLRRYDPSKEW
ZEBRAFISH TLSDNEGDWSILPLLPRLSSQ...FGKNSSWIMFLEEDTRV.KLQKLHEVLKKFDRRKEW
FRUITFLY VMHELFNSWTMLDALPHLRAQARVLGARTEWIIWCQHNTRVSSLRGLLEQLRRQNPRELA
STARFISH QQYEFSGVWTILPVLPKLNED...FGQ.AKWLFFCEEQTRV.SVHGLLQVLTKYNPQQEW
N-GT-like Domain
160 170 180 190 200 210
HUMAN FLGKALHDEEATIIHHYAFSENPTVFKYPDFAAGWALSIPLVNKLTKRL....KSESLKS
MOUSE FLGKALYDEESTIIHHYAFSENPTVFKYPDFAAGWALSIPLVNKLAKRL....KSEALKS
ZEBRAFISH FLGKPLHDEESTIIHHYAFSENPTAFEYPDFSAGWALSIPLINRLASKI....EEEPLKS
FRUITFLY FYGHALYDAEATIIHHFSNYKDPQRFPYPMLSAGVVFTGALLRRLADLVAPSGQNITVHS
STARFISH FLGRALQDRAASVIHHYRFHDDPSKFSYPDFEAGWLISTGLLRGLADRW....ETEEHRM
220 230 240
HUMAN DFTIDLKHEIALYIWDK...........GGGPPLTPVPEFCT......NDVDFY......
MOUSE DFTIDLKHEIALYIWDK...........GGGPALTPVPEFCT......EDVDPR......
ZEBRAFISH DFTIDLKHEVALYIWED...........GKGPRLTGVPELCTL..AEHNKRAQS......
FRUITFLY DFSIDASHELARFIFDNVSPDPHISTPISGGILLKSASYICSTPTSVPNRKLPC......
STARFISH DFSIDVKHELAYYILND...........GNGTRLTALPALCAG..NVEENPAPRSDSEIR
Linker
250 260 270 280
HUMAN ....................CATTFHSFLPLCRKPVKKKDIFVAVKTCKKFHGDRIPIVK
MOUSE ....................CVTTFHSFLPLCGVPVKKEEIFVAVKTCKKFHADRIPIVK
ZEBRAFISH ....................CATTVSSHSPLCGEPVKIENIFVAVKTCKKFHSDRVPVVK
FRUITFLY ..LLHAQPEEPLTLGQRRNGCEHTTGSH...........IYFAIKTCAKFHKERIPIIE
STARFISH KALNKAIRDGP....QEEDGCVSAHPKGLPTCAEAVLKEDILFAVKTCKKFHKDRVPIVQ
290 300 310 320 330 340
HUMAN QTWESQASLIEYYSDYTENSIPTVDLGIPNTDRGHCGKTFAILERFLNR..SQDKTAWLV
MOUSE KTWAAQASLIEYYSDYAETAIPTVDLGIPNTDRGHCGKTFAILEKFLNH..SHNKISWLV
ZEBRAFISH KTWGKQASLLEYYSDYADPSIPTINLGVPNTERGHCGKTFAILRRFLSS..HVPRTDWLL
FRUITFLY RTWAADARNRRYYSDVADVGIPAIGTGIPNVQTGHCAKTMAILQLSLKDIGKQLDIRWLM
STARFISH QTWGKHTSHIVFVSDVQDDTIPTIASGVPNTERGHCGKLFAIFEMFNSK.PEYLKYSWLV
C-GT Domain
350 360 370 380 390
HUMAN IVDDDTLI...........SISRLQHLLSCYDSGEPVFLGERYGYG.LGTG.GYSYITGG
MOUSE IVDDDTLI...........SISRLRHLLSCYDSSDPVFLGERYGYG.LGTG.GYSYVTGG
ZEBRAFISH IVDDDTLI...........SLPRLQALLSCYESSEPLCLGERYGYG.LGQG.GYSYITGG
FRUITFLY LVDDDTLLSLHLIHTHLPTSVPRVSALLCRHNATELVYLGQRYGYR.LHAPDGFNYHTGG
STARFISH VADDDTIL...........SVARLRALLSCYNAKKLVFLGERYGYGHLKLGWGYDYLTGG
400 410 420 430 440 450
HUMAN GGMVFSREAVRRLLASKCRCYSNDAPDDMVLGMCFSGLGIPVTHSPLFHQARPVDYPKDY
MOUSE GGMVFSREAIRRLLVSSCRCYSNDAPDDMVLGMCFSGLGVPVTHSPLFHQARPVDYPKDY
ZEBRAFISH GGMLFSREAVQLLLSSGCNCYSNDAPDDMVLGMCLNSLRVPVTHSPLFHQARPEDYARDF
FRUITFLY AGIVLSLPLV.RLIVQRCSCPSASAPDDMILGYCLQALGVPAIHVAGMHQARPQDYAGEL
STARFISH GGMIFSRAGIQQLLATGCKCNKDEDPDDMIFGMCTKRHDMPITHSPLFHQARPSDYAVGY
460 470 480 490
HUMAN LSHQVPISFHKHWNIDPVKVYFTWLA......PSDEDKARQETQKGF......REEL...
MOUSE LAHQIPVSFHKHWHIDPVKVYLTWLA......PSEEDQATQETQKDP......REEL...
ZEBRAFISH LSHQTPISFHKHWNIDPIAVFNKWL.........KNEKHTEGLQKST......KEEL...
FRUITFLY LQLHAPLTFHKFWNTDPEHTYRRWLGGSMVNRSAPLAAHKEQPAAGPLHMTMGRHSLAAG
STARFISH LQHQTPVSFHKHLNADPLAVYRDWFK......HADEVDSRTHGAQGE......REEL...

Figure S2

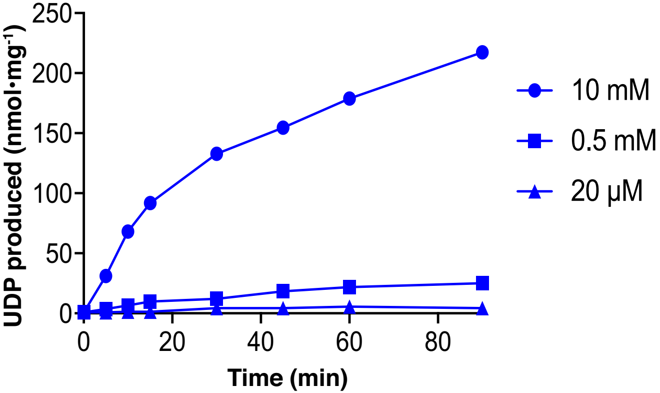

250
200
150
100
50
0
UDP produced (nmol·mg-1)
0
20
40
60
80
Time (min)
10 mM
0.5 mM
20 µM

Figure S3

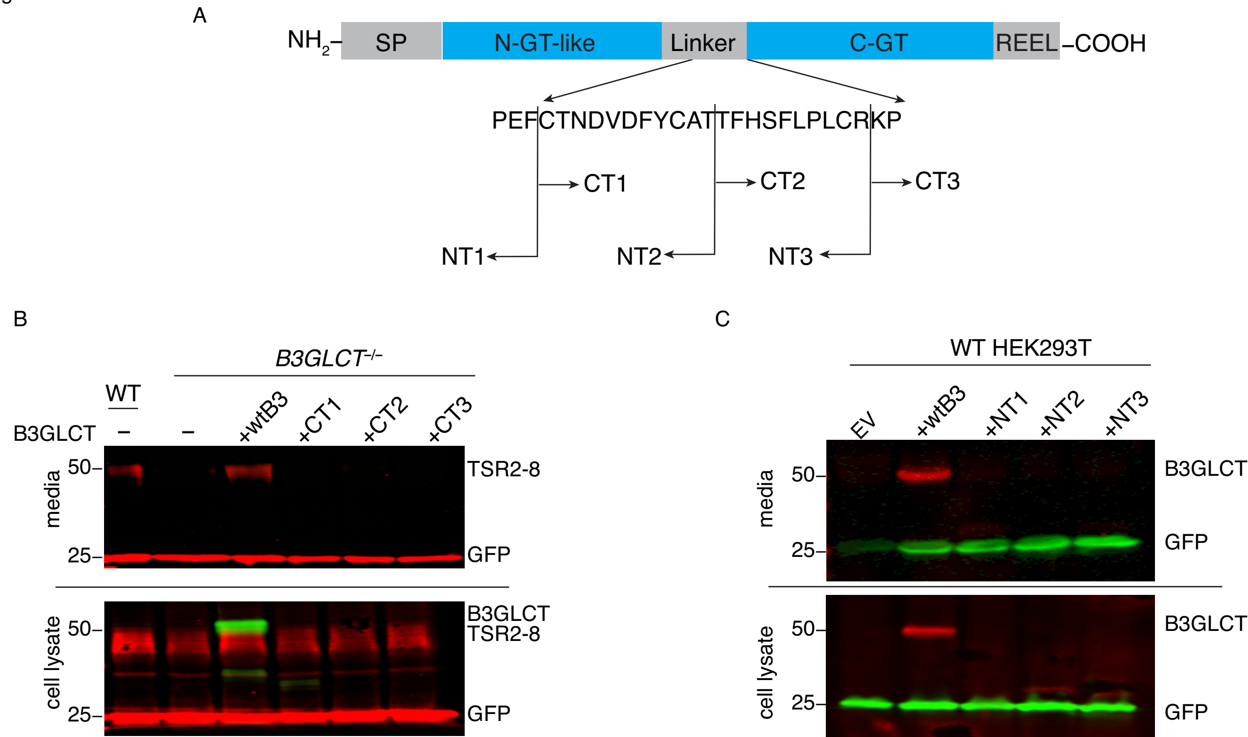
A
NH2–
SP
N-GT-like
Linker
C-GT
REEL
–COOH
PEFCTNDVDFYCATTFHSFLPLCRKP
CT1
CT2
CT3
NT1
NT2
NT3
B
B3GLCT-/-
WT
B3GLCT
–
–
+wtB3
+CT1
+CT2
+CT3
media
50–
25–
TSR2-8
GFP
cell lysate
50–
25–
B3GLCT
TSR2-8
GFP
C
WT HEK293T
EV
+wtB3
+NT1
+NT2
+NT3
media
50–
25–
B3GLCT
GFP
cell lysate
50–
25–
B3GLCT
GFP

Figure S4

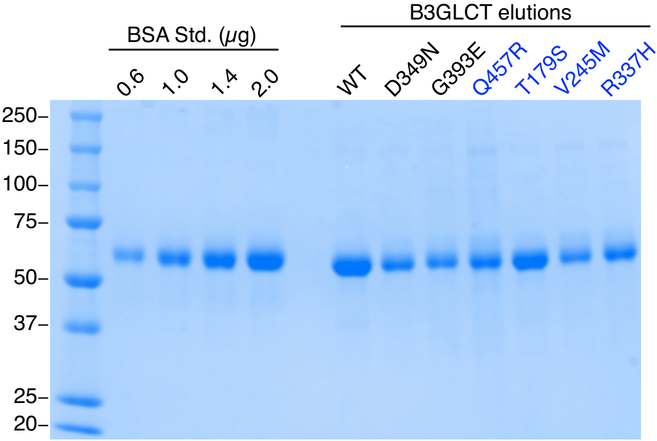

BSA Std. (μg)
0.6
1.0
1.4
2.0
B3GLCT elutions
WT
D349N
G393E
Q457R
T179S
V245M
R337H
250–
150–
100–
75–
50–
37–
25–
20–

Figure S5

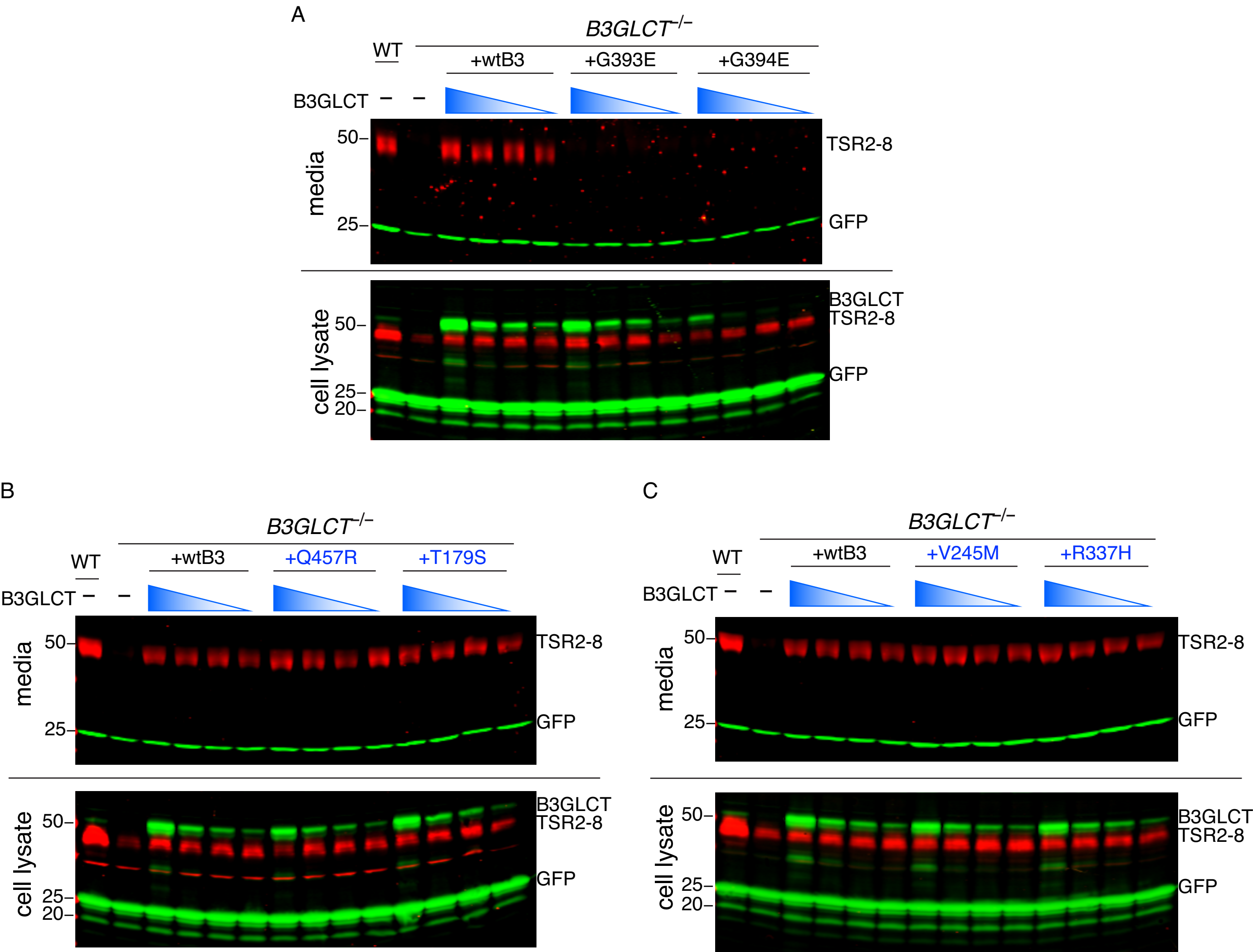

A
B3GLCT−/−
WT
+wtB3
+G393E
+G394E
B3GLCT
media
50
25
TSR2-8
GFP
cell lysate
50
25
20
B3GLCT
TSR2-8
GFP
B
B3GLCT−/−
WT
+wtB3
+Q457R
+T179S
B3GLCT
media
50
25
TSR2-8
GFP
cell lysate
50
25
20
B3GLCT
TSR2-8
GFP
C
B3GLCT−/−
WT
+wtB3
+V245M
+R337H
B3GLCT
media
50
25
TSR2-8
GFP
cell lysate
50
25
20
B3GLCT
TSR2-8
GFP

FigureS6

A

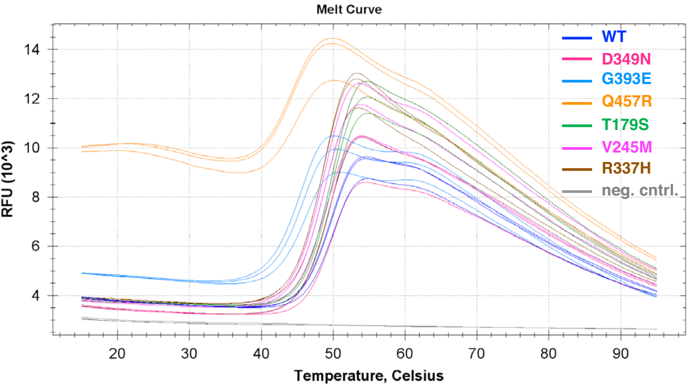
Melt Curve
RFU (10^3)
Temperature, Celsius
WT
D349N
G393E
Q457R
T179S
V245M
R337H
neg. cntrl.
14
12
10
8
6
4
20
30
40
50
60
70
80
90

B

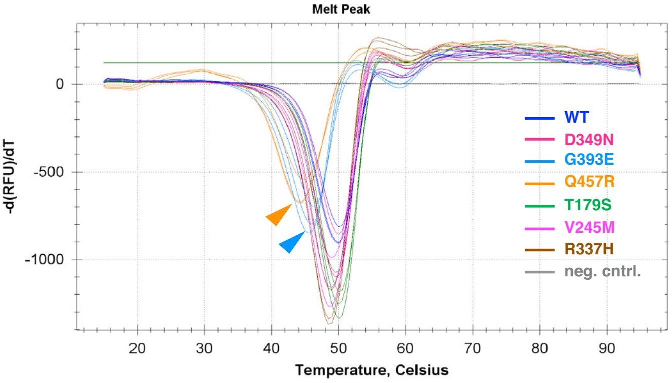
Melt Peak
-d(RFU)/dT
Temperature, Celsius
WT
D349N
G393E
Q457R
T179S
V245M
R337H
neg. cntrl.
0
-500
-1000
20
30
40
50
60
70
80
90

Figure S7

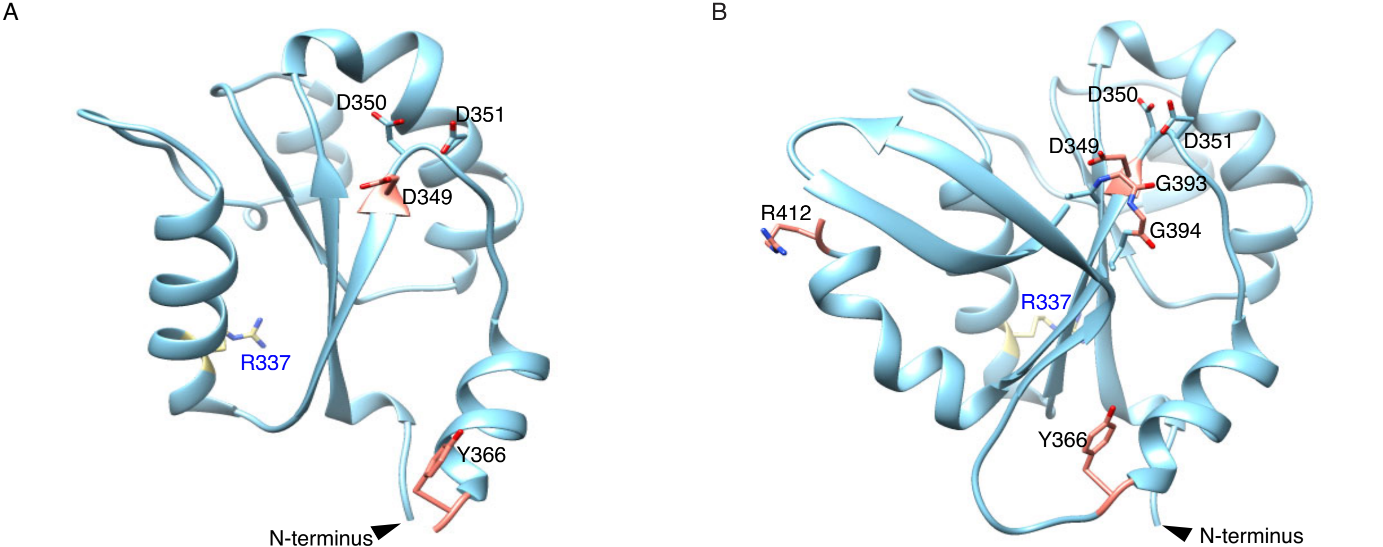
A
D350
D351
D349
R337
Y366
N-terminus
B
D350
D349
D351
G393
R412
G394
R337
Y366
N-terminus

Figure S8

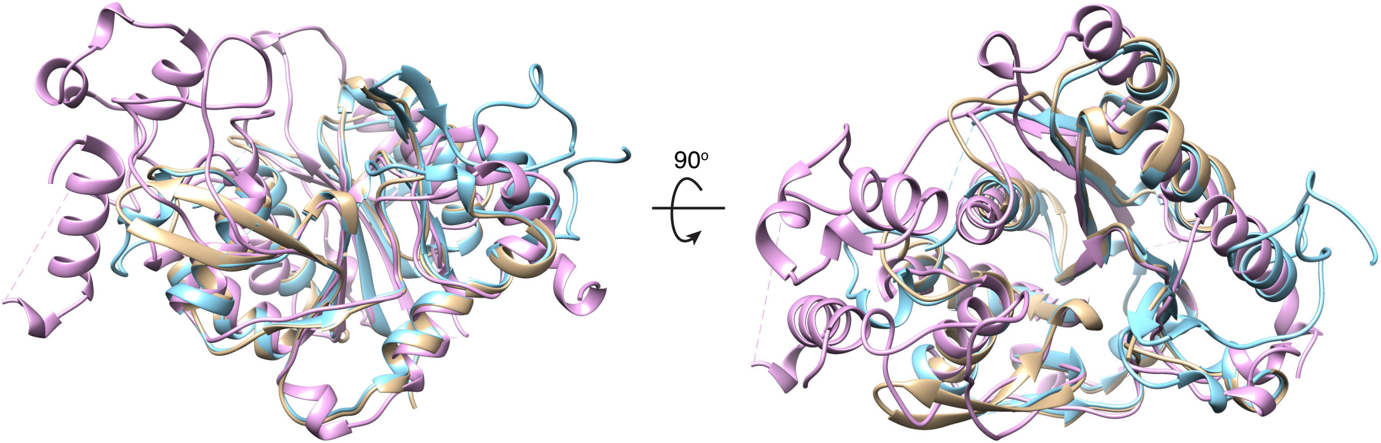

Figure S9

```
B3GNT2_HUMAN      ---------------------------------------------MS-VGRRRIKLLGILM  15
B3GLCT_HUMAN      MRPPACWWLLAPPALLALLTCSLAFGLASEDTKKEVKQSQDLEKSGISRKNDIDLKGIVF  60
MFNG_MOUSE        ------------------------------------------------------------  0

B3GNT2_HUMAN      MANV----FIYFIMEVSKSS--------S-----------QEKNGKGEVIIPKEKFWKIS  52
B3GLCT_HUMAN      VIQSQSNSFHAKRAEQLKKSILKQAADLTQELPSVLLLHQLAKQEGAWTILPLLPHFSVT  120
MFNG_MOUSE        ------------------------------------------------------------  0

B3GNT2_HUMAN      TPPEAYWNREQE-----------KLNRQYNPILSMLTNQTGEAGRLSNISHLNYCE----  97
B3GLCT_HUMAN      YSRNSSWIFFCEEETRIQIPKLLETLRRYDPSKEWFLGKALHDEEATIIHHYAFSENPTV  180
MFNG_MOUSE        ------------------------------------------------------------  0

B3GNT2_HUMAN      ---------------------PDLRVTSVVTGFN-----------------NLPDRFKD-  118
B3GLCT_HUMAN      FKYPDFAAGWALSIPLVNKLTKRLKSESLKSDFTIDLKHEIALYIWDKGGGPPLTPVPEF  240
MFNG_MOUSE        ----------------------------------------MHCRLFRGMAGAL---FTLL  17
                                                                  .

B3GNT2_HUMAN      --FLLYLRCRN--------YSLLIDQPDKCAK---KPFLLLAIKSL-TPHFARRQAIRES  164
B3GLCT_HUMAN      CTNDVDFYCATT-----FHSFLPL-----CRKPVKKKDIFVAVKTCKKFHGDRIPIVKQT  290
MFNG_MOUSE        CVGLLSLRYHSSLSQRMIQGALRLNQRNPGPLELQLGDIFIAVKTTWAFHRSRLDLLLDT  77
                      : :   .          * :              :::*:*:    *  *   : ::

B3GNT2_HUMAN      WGQESNAGNQTVVRVFLLGQTPPEDNHPDLSDMLKFE---SEKHQDILMWNYRDTFFNLS  221
B3GLCT_HUMAN      WESQAS------LIEYYSDYTENSIPTVDLG----IPN--TDRGHCGKTFA---------  329
MFNG_MOUSE        WVSRIR------QQTFIFTDSPDERLQERLGPHLVVTNCSAEHSHPALSCK---------  122
                  * ..           :    :  .     *.    .    ::: :

B3GNT2_HUMAN      LKEVLFLRWVSTSCPDTEFVFKGDDDVFVNTHHILNYLNSLSKTKAKDLFIGDVIHNAGP  281
B3GLCT_HUMAN      ----ILERFLNRSQDKTAWLVIVDDDTLISISRLQHLLSCYD--SGEPVFLGERYGY---  380
MFNG_MOUSE        ----MAAEFDAFLVSGLRWFCHVDDDNYVNPKALLQLLKTFP--QDRDVYVGKPSLNRPI  176
                      :  .:         :.   ***  :.   : : *.     . . :::*.

B3GNT2_HUMAN      HRDKKLKYYIPEVVYSGLYPPYAGGGGFLYSGHLALRLYHITDQVHL-------YPIDDV  334
B3GLCT_HUMAN      ------------GLGTGGYSYITGGGGMVFSREAVRRLLASKCRC-Y-----SNDAPDDM  422
MFNG_MOUSE        HASEL---QSKNRTKLVRFWFATGGAGFCINRQLALKMVPWASGSHFVDTSALIRLPDDC  233
                                    :   :**.*:  . . . ::                    **

B3GNT2_HUMAN      YTGMCLQ-KLGLVPEKHKGFR----TFDIEEKN---KNNICSYVDL--------ML----  374
B3GLCT_HUMAN      VLGMCF-SGLGIPVTHSPLFHQARPVDY--PKDYLSHQVPISFHKHWNIDPVKVYFT-WL  478
MFNG_MOUSE        TVGYIIECKLGGRLQPSPLFHSHLETLQLLGAAQLPEQVTLSYGVF----EGKLNVIKLP  289
                    *  :   **        *:    .          .:   *:            .

B3GNT2_HUMAN      -------VHSRKPQEMIDIWSQLQSAHLKC------   397
B3GLCT_HUMAN      APSDEDKARQETQKGFREEL----------------   498
MFNG_MOUSE        GPF----SHEEDPSRFRSLHCLLYPDTPWCPLLAAP   321
                          :..  . : .
```

Figure S10

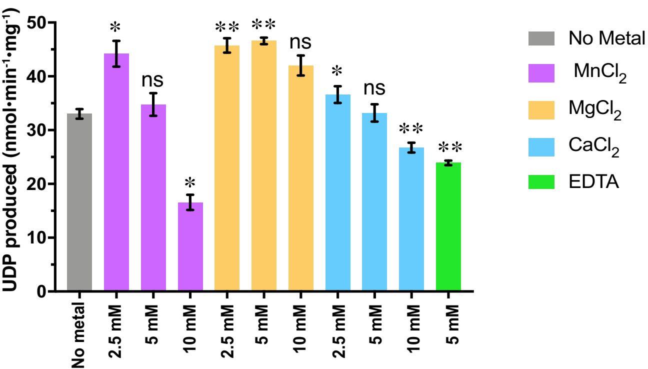

UDP produced (nmol•min-1•mg-1)
50
40
30
20
10
0
*
ns
*
**
**
ns
*
ns
**
**
No metal
2.5 mM
5 mM
10 mM
2.5 mM
5 mM
10 mM
2.5 mM
5 mM
10 mM
5 mM
No Metal
MnCl2
MgCl2
CaCl2
EDTA

Figure S11

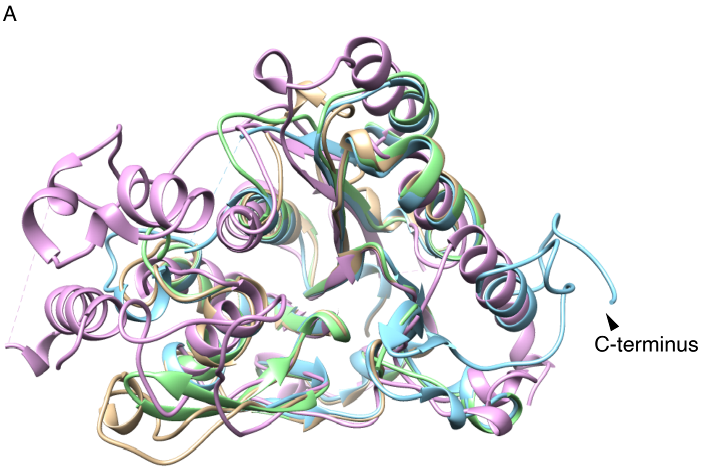
A
C-terminus

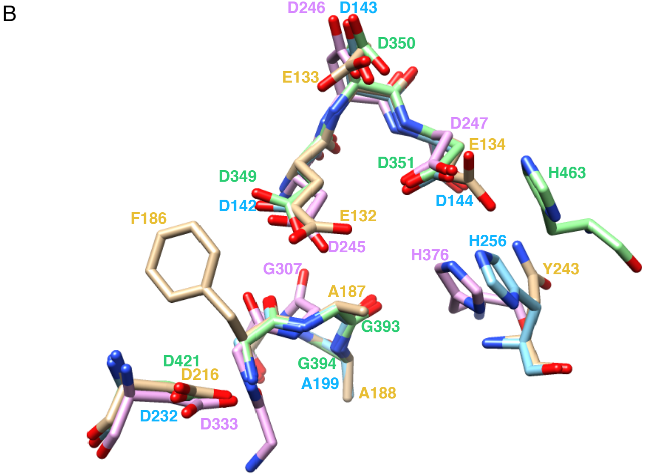
B
D246
D143
D350
E133
D247
E134
D351
H463
D349
D142
D144
E132
F186
D245
H256
H376
Y243
G307
A187
G393
G394
D421
D216
A199
A188
D232
D333
